# Transcriptome profiling reveals dysregulation of inflammatory and protein synthesis genes in PCOS

**DOI:** 10.1038/s41598-024-67461-4

**Published:** 2024-07-18

**Authors:** Xilian Li, Biao Gao, Bingsi Gao, Xin Li, Xian Xia

**Affiliations:** 1 Obstetrics and Gynecology Hospital of Fudan University, Shanghai, 200011 China; 2 Teaching and Research Support Center, Naval Medical University, Shanghai, 200433 China

**Keywords:** PCOS, Co-DEGs, Genomic analysis, Genetics, Diseases, Molecular medicine

## Abstract

To analyze the differential expression genes of polycystic ovary syndrome (PCOS), clarify their functions and pathways, as well as the protein–protein interaction network, identify HUB genes, and explore the pathological mechanism. PCOS microarray datasets were screened from the GEO database. Common differentially expressed genes (co-DEGs) were obtained using GEO2R and Venn analysis. Enrichment and pathway analyses were conducted using the DAVID online tool, with results presented in bubble charts. Protein–protein interaction analysis was performed using the STRING tool. HUB genes were identified using Cytoscape software and further interpreted with the assistance of the GeneCards database. A total of two sets of co-DEGs (108 and 102), key proteins (15 and 55), and hub genes (10 and 10) were obtained. The co-DEGs: (1) regulated inflammatory responses and extracellular matrix, TNF, and IL-17 signaling pathways; (2) regulated ribosomes and protein translation, ribosome and immune pathways. The key proteins: (1) regulated inflammation, immunity, transcription, matrix metabolism, proliferation/differentiation, energy, and repair; (2) regulated ubiquitination, enzymes, companion proteins, respiratory chain components, and fusion proteins. The Hub genes: (1) encoded transcription factors and cytokines, playing vital roles in development and proliferation; (2) encoded ribosomes and protein synthesis, influencing hormone and protein synthesis, associated with development and infertility. The dysregulated expression of inflammation and protein synthesis genes in PCOS may be the key mechanism underlying its onset and progression.

## Introduction

Polycystic ovary syndrome (PCOS) is a prevalent gynecological reproductive disorder affecting 4–7% of women globally, characterized by prolonged anovulation, hyperandrogenism, and polycystic ovaries. These features are not only closely associated with infertility but may also significantly impact the quality of life and mental health^1^. Although various treatment methods have been employed, there remains room for improvement in their efficacy and approaches^2^. Elucidating the intricate pathophysiological mechanisms underlying PCOS is crucial for identifying more effective therapies, Which represents an urgent biomedical research goal with significant clinical implications.

In recent years, rapid advances in genetic analysis have led to significant progress in deciphering the genetic architecture of PCOS pathogenesis. Various PCOS-associated genetic loci have been identified, including gene mutations, polymorphisms^3,4^, and potential epigenetic effects^5^. These genetic variations implicate critical biological processes like androgen metabolism and insulin signaling, providing vital clues to unraveling PCOS pathophysiology^5,6^. However, the complex multifactorial etiology of PCOS poses challenges for elucidation by single gene studies alone. Heterogeneous genetic backgrounds among populations lead to inconsistent findings, and gene regulatory networks underlying PCOS remain incompletely defined^7^. Therefore, a holistic, systematic approach is imperative, aggregating extensive sample datasets to uncover common PCOS-associated differentially expressed genes (co-DEGs), delineate their correlations with clinical presentation, and lay a scientific foundation for precise diagnosis and treatment^5^.

This study utilized publicly available information from the GEO database to screen and analyze specific gene expression microarray datasets to identify differentially expressed genes (DEGs) associated with PCOS. The aim was to identify co-DEGs in PCOS patients, explore their roles in biological functions and signaling pathways, and analyze the characteristics of protein–protein interaction (PPI) networks and key genes. We hoped to elucidate further the pathological mechanisms underlying PCOS through these analyses, providing a scientific basis for developing more precise targeted therapies.

## Materials and methods

This study is a systematic bioinformatics analysis of publicly available microarray datasets from the Gene Expression Omnibus (GEO) database. The aim is to identify differentially expressed genes (DEGs) and key pathways involved in the pathogenesis of polycystic ovary syndrome (PCOS) by integrating multiple datasets and performing functional enrichment analysis, pathway analysis, and protein–protein interaction (PPI) network analysis. The study does not involve direct human participants or animal experiments, as it relies solely on the computational analysis of pre-existing, publicly available gene expression data.

### Materials

On September 24, 2023, publicly available microarray datasets, which are the original data uploaded by researchers and then corrected, quality controlled, organized, standardized, and normalized by GEO, were retrieved and selected from the GEO (Gene Expression Omnibus database) DataSets of the National Center for Biotechnology Information (NCBI), to obtain Transcriptomic profiling data of human reproductive tissue samples.

### Data acquisition

#### Search strategy

Primary keywords: PCOS, polycystic ovary syndrome; Data type: Datasets suitable for GEO2R analysis; Species limited to: Humans (Homo sapiens).

#### Search query

((PCOS) OR (“polycystic ovary syndrome”)) AND “Homo sapiens”[porgn:txid9606] AND (GEO2R).

### Data criteria

Inclusion criteria: PCOS gene differential analysis; Samples from human ovarian tissue, endometrium; Control group present; Data analyzed by GEO2R for differentially expressed genes (DEGs) that meet Padj < 0.05 and |log2FC|> 1. Exclusion criteria: Non-compliant samples, including in vitro cultured cells; Non-English studies; Duplicated or retracted studies; Non-coding RNA genes without corresponding Symbols; Incomplete data; Post-intervention gene expression.

Selection basis: Reference to inclusion and exclusion criteria based on the title, abstract, and published papers of the research project; Specifics of DEGs verified by GEO2R.

### Data size

In the initial analysis, datasets from 64 studies were included. Among them, 5 groups of data from 4 study datasets met the criteria for further analysis (containing 24, 6, 8, 12, and 6 samples, respectively). Ultimately, 3 groups of data (with 6, 8, and 6 samples, respectively) yielded 2 sets of common differentially expressed genes (108 and 102 genes, respectively).

### Analysis methods

GEO2R analysis: Each microarray study was reviewed to select eligible studies according to the inclusion criteria. The GEO2R online analysis platform was utilized to verify these chip array data, analyze their DEGs, and identify the platforms used for gene chip array data.

Venn analysis: Preliminary selected datasets’ common DEGs (co-DEGs) were obtained through Venn diagram intersection analysis. The intended gene chips for analysis were selected, followed by detailed analysis and verification to get the final co-DEGs for functional annotation, pathway analysis, and HUB gene analysis.

**Figure 1:**
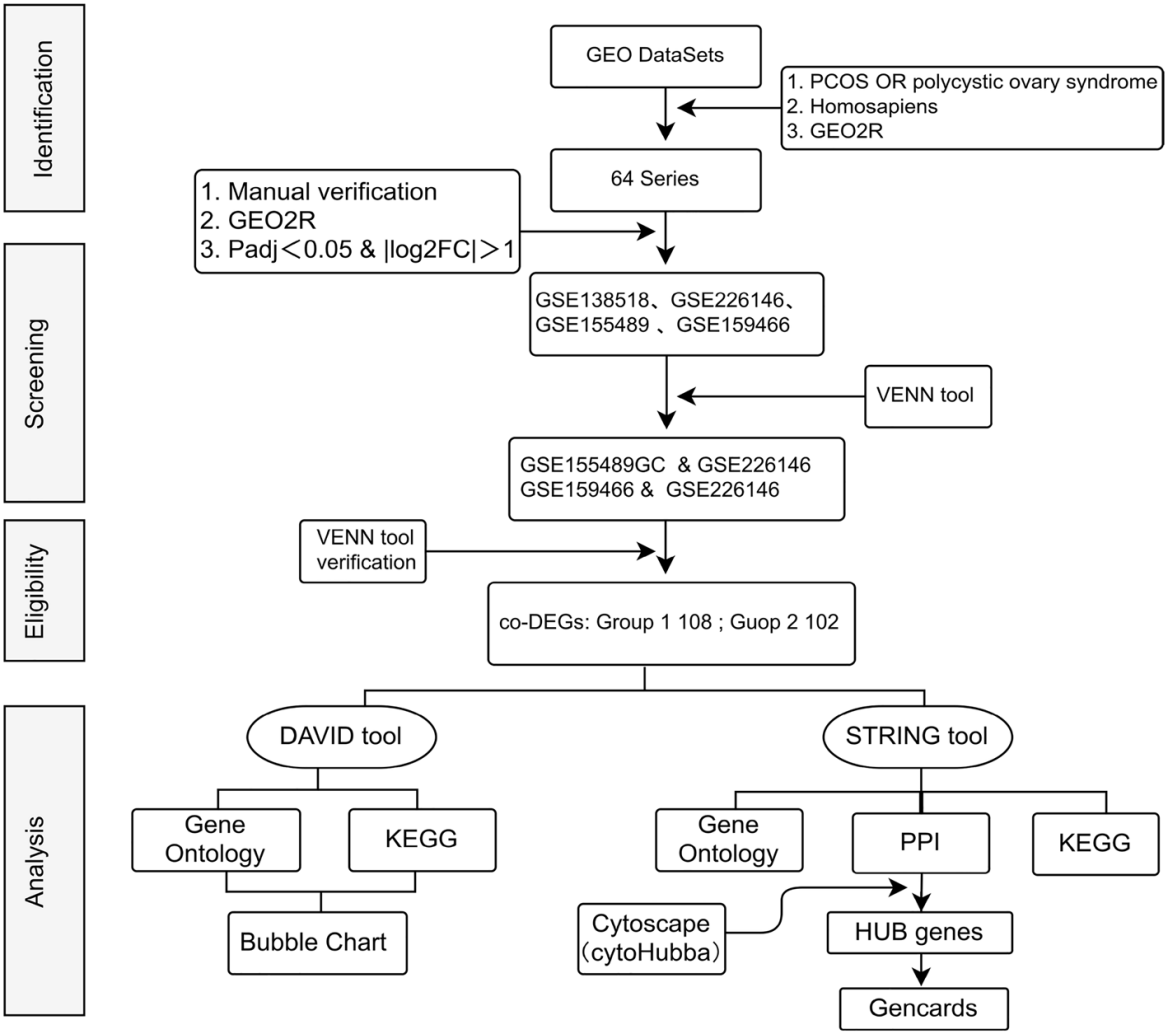
Data acquisition and analysis flowchart.

Co-DEGs analysis: DAVID and STRING online network systems were used for functional enrichment, KEGG pathway, and PPI (Protein–Protein Interaction) analysis. Cytoscape software (3.8.2, cytoHubba plugin) was employed to identify HUB genes and interpret them using GENCARDS. The data acquisition and analysis process is depicted in Fig. 1.

### Ethics statement

The research protocol was reviewed and approved by the research ethics committee of the obstetrics and gynecology hospital of Fudan university. All aspects of thish study were conducted in accordance with guidelines of the obstetrics and gynecology hospital of Fudan university.

## Results

**Figure 2:**
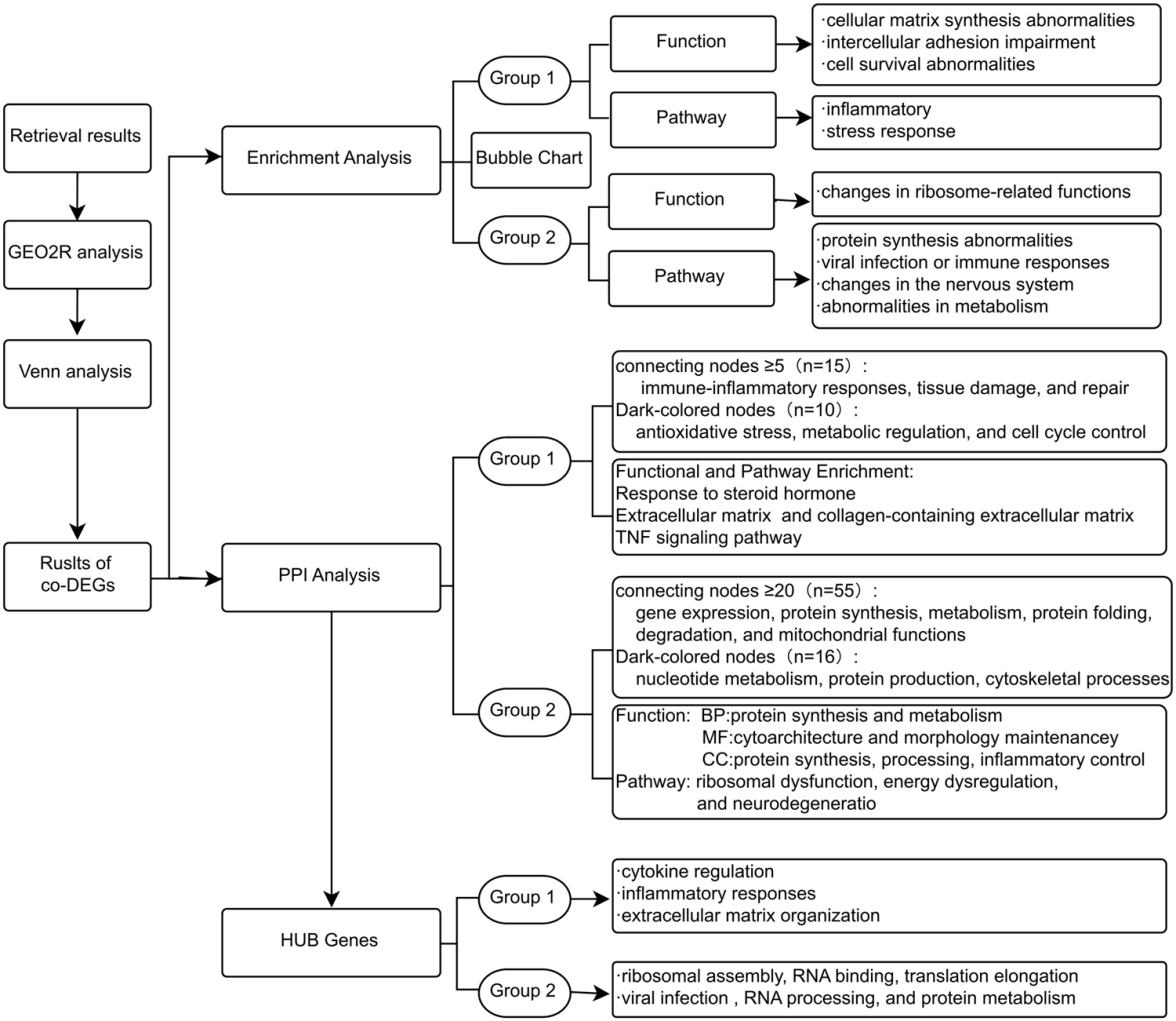
Work flow chart of analysis and results. Grop1,GSE155489GC, GSE226146; Grop2,GSE159466,GSE226146.

A workflow chart is made to visually show the research ideas and results (Fig. 2).

A search yielded 64 studies, each analyzed and screened to identify gene chip array data suitable for this research, which led to the selection of data from four gene chip arrays, GSE138518, GSE226146, GSE155489, and GSE159466, encompassing five groups of data (GSE155489 includes two sets of data: one from oocytes and the other from cumulus granulosa cells). The GEO2R online tool was utilized to analyze these original datasets.

### GEO2R analysis

#### GSE138518

**Figure 3:**
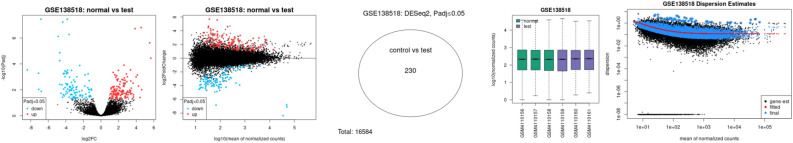
GEO2R analysis of GSE138518.

Release Date: October 8, 2019. Tissue: Ovarian Granulosa Cells (GC). Research content: Transcriptome analysis of ovarian tissues from 12 PCOS patients and 12 normal controls. Research Design: RNA sequencing technology was applied to compare ovarian granulosa cells from PCOS patients with those from normal individuals^8^, with related literature already published^9^. GEO2R was used to analyze the original data to identify Differentially Expressed Genes (DEGs). A total of 16,584 genes were analyzed, of which 230 met the criteria of *P*adj < 0.05, and 225 had |log2FC|> 1 (Fig. 3).

### GSE226146

**Figure 4:**
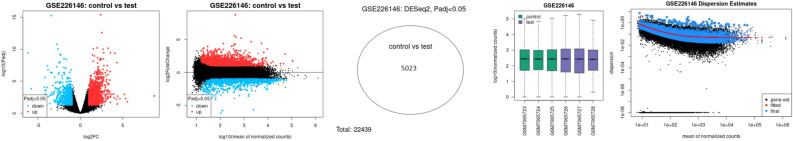
GEO2R analysis of GSE226146.

Release date: March 6, 2023. Tissue: Endometrium. Title: LncRNAs and mRNA expression profiling in the endometrium of PCOS patients undergoing in vitro fertilization-embryo transfer. Research content: This study conducted expression profiling analysis based on high-throughput RNA sequencing to compare the expression levels of long non-coding RNAs (lncRNAs) and messenger RNAs (mRNAs) in the endometrial tissue between patients with polycystic ovary syndrome (PCOS) and individuals with normal ovulation, followed by a comparative analysis of the two groups. Research design: Comparative analysis of RNA-seq data for gene expression in 3 cases of PCOS and 3 normal individuals^10^. DEGs were identified through GEO2R analysis of the original data, with 24,239 genes. Among these, 5,023 met the criteria of *P*adj < 0.05, and 2,557 had |log2FC|> 1 (Fig. 4).

### GSE155489

Release Date: September 8, 2020. Tissue: Oocytes (OO) and Cumulus Granulosa Cells (GC). Title: Transcriptomic analysis of oocytes and cumulus cells reveals comprehensive molecular characteristics of polycystic ovary syndrome. Research content: This study involves transcriptomic analysis of oocytes and cumulus GCs from PCOS patients to elucidate the molecular characteristics of PCOS and identify its pathogenic factors. Research design: Oocytes and GCs were obtained from PCOS patients and matched non-PCOS female controls for RNA sequencing analysis to investigate the transcriptional characteristics of the oocytes and GCs^11^.

**Figure 5:**
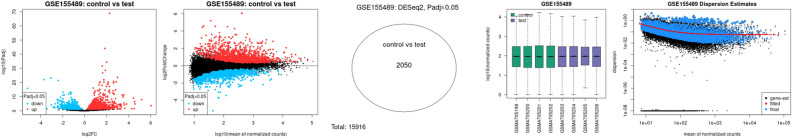
GEO2R analysis of cumulus granulosa cells in GSE155489.

Data from cumulus granulosa cells (cumulus GCs) of 4 pairs of PCOS and control groups were selected, and original data were analyzed using GEO2R to find DEGs. A total of 15,916 genes were identified, among which 2,050 met the criteria of *P*adj < 0.05, and 878 had |log2FC|> 1 (Fig. 5).

**Figure 6:**
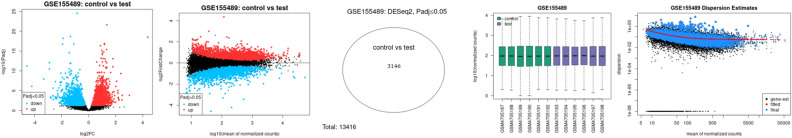
GEO2R analysis of oocyte cells in GSE155489.

Data from follicular cells (oocytes) of 6 pairs of PCOS and control groups were selected, and original data were analyzed using GEO2R to find DEGs. A total of 13,416 genes were identified, among which 3,146 met the criteria of *P*adj < 0.05, and 864 had |log2FC|> 1 (Fig. 6).

### GSE159466

**Figure 7:**
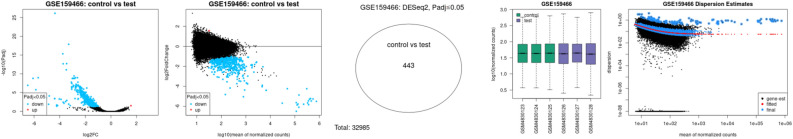
GEO2R analysis of GSE159466 Data.

Release date: March 11, 2021. Tissue: Follicle fluid. Title: Aberrant expression of exosomal long non-coding RNAs in patients with polycystic ovary syndrome follicle fluid. Research content: Expression profiling analysis was conducted using high-throughput sequencing technology for lncRNA analysis. Total RNA was extracted from the follicular fluid exosomes of the test group (PCOS patients) and the control group (infertile patients without PCOS), respectively. Subsequently, the RNA samples underwent library preparation, sequencing, and data processing to identify differentially expressed lncRNAs between the two groups. Research Design: Expression profiling analysis was conducted using high-throughput sequencing for non-coding RNA analysis^12^*,* with related literature published^13^. A comparison of DEGs between the test group (*n* = 3) and control group (*n* = 3) was performed, identifying 443 genes that met the criteria of *P*adj < 0.05 and |log2FC|> 1 (Fig. 7).

### Venn analysis

**Figure 8:**
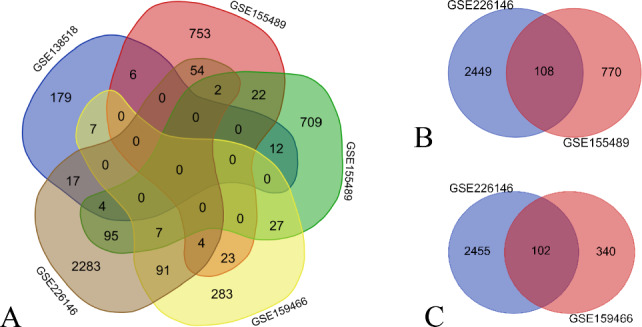
Venn analysis for the identification of common DEGs. (**A**): Venn analysis of 5 data sets (GSE138518, GSE226146, GSE155489, GSE155489GC, GSE159466) from 4 gene chips; (**B**): Venn analysis of 2 gene data sets (GSE226146, GSE155489GC); (**C**): Venn analysis of 2 gene data sets (GSE226146, GSE159466).

An online tool (https://bioinformatics.psb.ugent.be/webtools/Venn/) was utilized to analyze the intersection of DEGs across four gene microarray datasets encompassing five groups. The results are depicted in Fig. 8A. Microarrays with fewer intersecting results were excluded from refining the analysis, focusing on GSE226146 (*n* = 6) and GSE155489GC (*n* = 8) (Fig. 8B). Additionally, DEGs from the GSE159466 (*n* = 6) and GSE226146 microarrays were analyzed separately (Fig. 8C), identifying intersecting DEGs, as listed in Table 1.

**Table 1:** Two groups of co-DEGs from 3 gene chips.

GSE	Count	Genes symbol
GSE155489GC GSE226146 (Group1)	108	CXCR4 LOC105372669 LINC01279 EPB41L4B ACSM3 COL1A1 VGLL3 ZNF22-AS1 TNXB LOC112268426 SDC2 CROCCP3 CLSTN2 INPP5J PCSK6 LOC112268447 RASSF4 SLC46A2 GJA9 RAB20 PTGS2 TRANK1 NR4A3 SOCS3 VWA3A PKP2 NPAS2 CXCL2 ABCA6 MIR6717 NR4A1 MXRA7 CEACAM21 P3H2 CRACD LOC102724428 THBS1 PID1 MOCOS LOC107984485 MTFP1 FILIP1L ICAM3 FOSB PFKFB4 CYB5A ANKRD18A ERRFI1 NME1 SIK1 IFIT3 WLS TGFB2 ANXA1 PRDX6 ATF3 COL15A1 SLC25A1 TNFRSF11B LRRC17 ETS2 LOC105373269 CDR2 BNIP3 KIAA1217 MAP3K8 STC1 STOM LDHA GAMT TUBA3D DUSP5P1 TRIB1 SPAG4 LAMA5 CDC7 GCNA LOC107985193 FBXO21 CBLN1 ARHGAP9 MAOB LOC107984131 JUNB LOC107986813 AEBP1 ACP3 LOC105376025 LOC112267956 AKAP5 HLA-L PORCN SHISA2 BCL3 CST2 TMEM141 ELL2 THSD4 PEG10 PRKXP1 RFLNB LOC100507472 PINX1 LOC105376216 CIART CBR1 BIRC3 AMZ1
GSE159466 GSE226146 (Group2)	102	RPS11 RPS17 RPL17-C18orf32 TMSB10 RPL19 UBB RPS5 RPL7A DAD1 RPL35 COL1A1 TPI1 RPS28 SERPING1 RPLP1 LDHB NME1-NME2 COX7A2 RPL32 RPS10-NUDT3 RAB20 RPL17 CDK16 RCN1 SF3B5 ARPC1A RPL24 IGFBP5 NME4 SMOC2 C1S MIR1282 RPLP0 SRP14 UQCR10 RPS10 RPL15 CFL1 RPL28 RPL18A THBS1 TUBA1C HSP90AB1 SSR2 RPL9 TFPI RPL35A MARCKSL1 RNA18SN5 RPL23A PSMA6 OST4 CYB5A GAPDH COX7C RPL38 RPS16 RPL10A GJA1 PRDX6 YWHAE TRIR RPS4X ATP5MJ MYL6 RPSA TUBA1B RPS2 ZYX H4C5 CALR RNA18SN2 LDHA SCD RPS26 RPL13AP5 GOS2 EEF1B2 RPL30 RPL36AL RPL26 NDUFB10 RPL36 RACK1 NME2 RPL21 ADAMTS1 RPS15A RPS12 UBA52 RPL27A PRDX2 RPL13A RPL41 ATP5F1B CETN2 TIMM13 CST2 RPS14 TMA7 EEF1G RPS27

### Enrichment analysis

Functional enrichment annotation and pathway enrichment analysis of the co-DEGs from the two groups were conducted using DAVID, STRING online tools, and Cytoscape software. Additionally, PPI and HUB gene analyses were performed, and the HUB genes were interpreted with the assistance of Gencards.

#### Function and pathway

Functional and pathway enrichment analyses were conducted using the DAVID online database (https://david.ncifcrf.gov/), which included selecting GO (Gene Ontology) functional annotations and KEGG (Kyoto Encyclopedia of Genes and Genomes) pathway enrichment to examine highly enriched DEG functions and annotations. The GO analysis encompasses biological process (BP), cellular component (CC), and molecular function (MF).

##### Group 1

**Figure 9:**
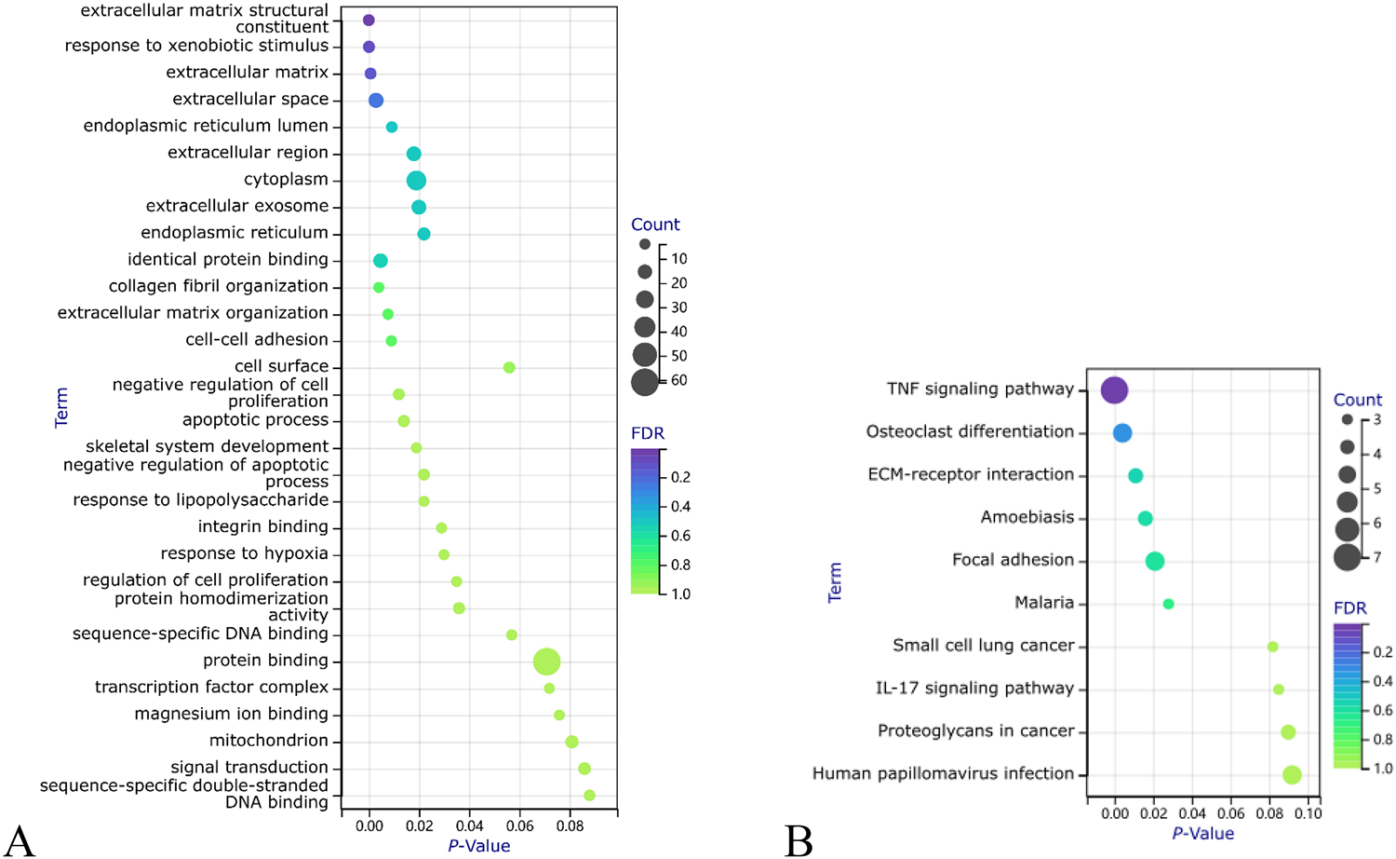
Bubble chart for functional and pathway enrichment of group 1**. (A**): Bubble chart of gene ontology (GO) functional enrichment; (**B**): Bubble chart of Kyoto Encyclopedia of Genes and Genomes (KEGG) pathway enrichment. FDR (false discovery rate): A measure used to control the rate of false-positive findings, considered effective when below 0.05. In GO analysis via DAVID, the Benjamini method calculates FDR values. Lower FDR values indicate more statistically reliable pathway enrichment. *P*-Value: Represents the significance of enrichment for each pathway. Typically, a threshold of 0.05 is set, meaning lower *P*-values denote more significant enrichment and a greater relevance to gene function. However, *P*-values alone cannot distinguish false positives and must be interpreted with the FDR. Count: The number of genes.

For the 108 DEGs obtained from the Venn analysis, the DAVID database recognized 102. During the functional enrichment analysis, the parameters were set to Count ≥ 4 and EASE (Enrichment Analysis Systematic Explorer) to 0.1, yielding 30 significant results (Table 2). The same 108 genes were subjected to KEGG analysis with the settings Count ≥ 3 and EASE 0.1, yielding 10 effective results (Table 3). The enrichment analysis results were visualized using the SangerBox online tool (http://vip.sangerbox.com/) (Fig. 9).

**Table 2:**
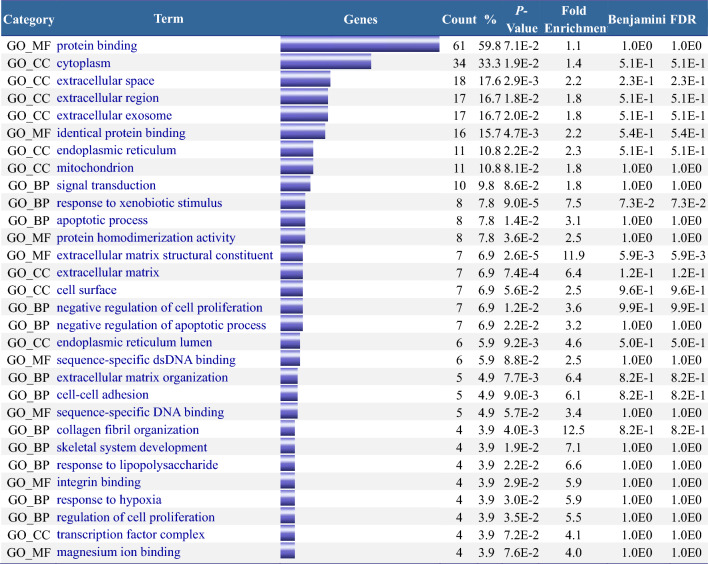
Functional enrichment analysis (DAVID) of 102 DEGs in group 1.

*BP* (biological process) refers to the molecular functions the gene products will likely perform. *CC* (cellular component) indicates the cellular environment where the gene products will likely be located. *MF* (molecular function) pertains to the biological processes in which the gene products may be involved. Category denotes the type of enriched function; Term provides a specific description of the function; Count represents the number of genes enriched for that function.

*P-value assesses the statistical significance of the results. The Benjamini value, derived from the Benjamini–Hochberg false discovery rate (FDR) correction for multiple hypothesis testing, reflects the expected proportion of false positives among all positive findings (i.e., those tested as significant), with smaller values indicating a more reliable association between the function term and the input genes. FDR represents the original false positive rate before multiple hypothesis testing adjustments, showing all tests’ expected proportion of false positives.*

**Table 3:**
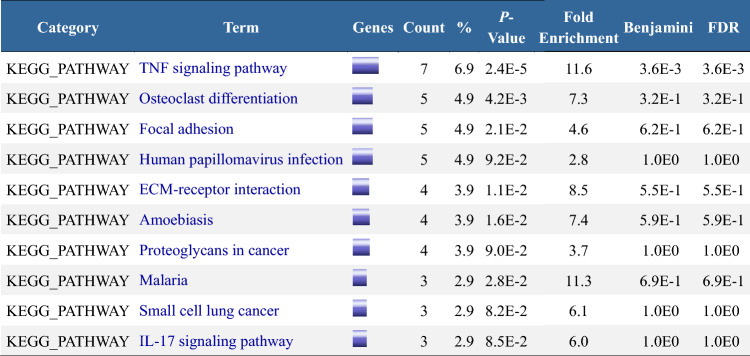
Pathway enrichment analysis (DAVID)of 102 DEGs in GSE155489GC and GSE226146 groups.

*KEGG* (Kyoto Encyclopedia of Genes and Genomes) is a database for pathway information. Category: The category of enriched functions. Term: Specific description of the function. Count: The number of genes enriched in the pathway.

*P-value: Assesses whether the results are statistically significant. Benjamini: The false discovery rate (FDR) was adjusted using the Benjamini–Hochberg method for multiple hypothesis testing. It reflects the expected proportion of false positives among all positives (i.e., those tested as significant). A lower value indicates a more reliable association between the function (term) and the input genes. FDR (false discovery rate): The original false discovery rate before multiple hypothesis testing adjustments, reflecting the expected proportion of false positives in all tests.*

The functional and pathway enrichment analyses provided a comprehensive understanding of the biological significance of the DEGs in PCOS. This approach helps identify these genes' critical biological processes, cellular components, and molecular functions and their association with known pathways. The integration of these findings with PPI and HUB gene analyses further elucidates the molecular mechanisms underlying PCOS, offering potential targets for therapeutic intervention and a deeper understanding of the disease pathology.

##### Group 2

**Figure 10:**
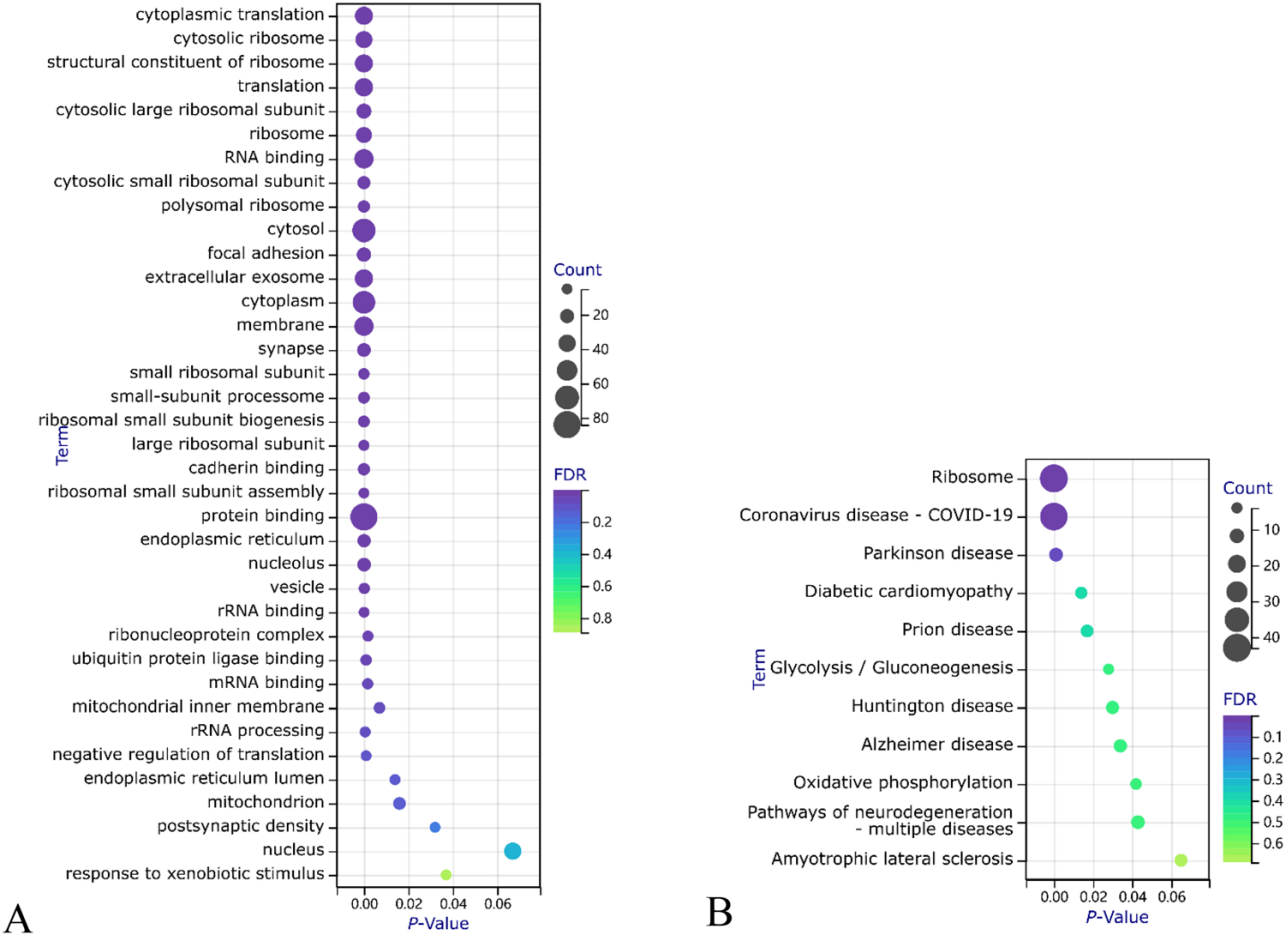
Bubble chart of functional and pathway enrichment for group 2. (**A**): GO Functional Enrichment Bubble Chart. (**B**): KEGG Pathway Enrichment Bubble Chart. FDR (False Discovery Rate): FDR indicates the expected proportion of false positive findings among all discoveries. An FDR value below 0.05 effectively controls the rate of false positives. In GO analysis using DAVID, the Benjamini method calculates the FDR values. The lower the FDR value, the more statistically reliable the pathway enrichment is. *P*-Value: Represents the significance of enrichment for each pathway. Typically, a *P*-value threshold of 0.05 is set, meaning the smaller the *P*-value, the more significant the enrichment, and the greater the relevance to gene function. However, the *P*-value alone cannot differentiate false positives; it should be interpreted with the FDR for a comprehensive assessment. Count: The count of genes.

For the 102 DEGs obtained from the Venn analysis, GO analysis was conducted using the DAVID online tool. The parameters were set to Count ≥ 5 and EASE (Enrichment Analysis Systematic Explorer) to 0.1, resulting in 37 significant findings (Table 4). The same 102 genes were subjected to KEGG analysis with the settings Count ≥ 4 and EASE 0.1, yielding 11 effective results (Table 5). The SangerBox online tool was employed to visualize the enrichment analysis results (Fig. 10).

**Table 4:**
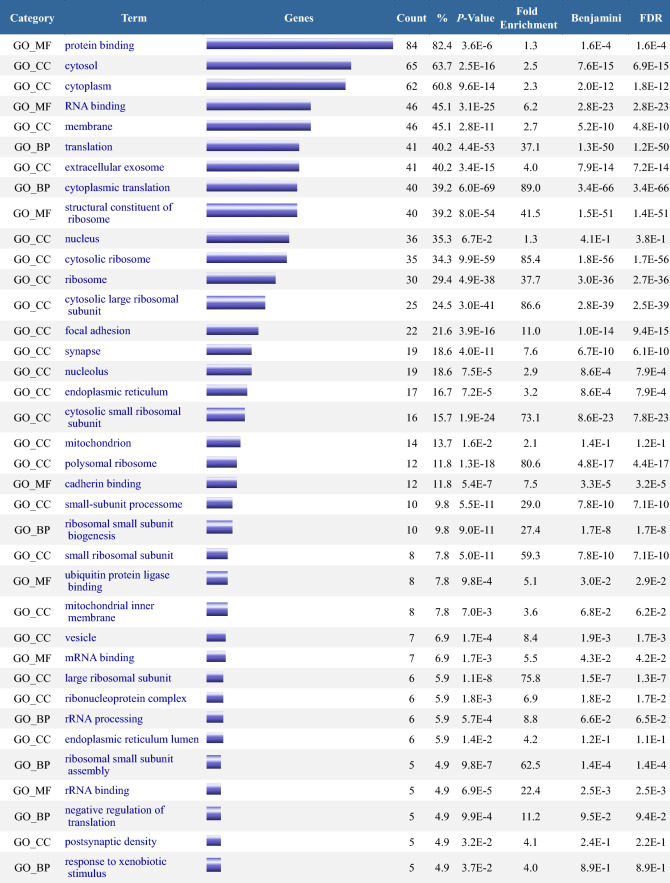
GO Annotation Enrichment Analysis (DAVID) of 102 DEGs in Group 2.

*BP* (Biological Process): Biological processes describe the molecular functions in which gene products are likely involved. *CC* (Cellular Component): Cellular localization indicates the cellular environment where gene products are potentially located. *MF* (Molecular Function): Molecular functions relate to the biological processes in which gene products may participate. Category: Category of enriched functions, highlighting the classification of genes in biological processes, cellular components, or molecular functions. Term: Specific functional description detailing the role of gene sets in particular biological processes, cellular components, or molecular functions. Count: The number of genes enriched in the specific function indicates the number of genes identified as differentially expressed within the specified functional term.

*P-value: Statistical significance assessment, calculated using the adjusted Fisher's exact test, used to evaluate whether the enrichment of a specific functional term is statistically significant. Benjamini: False discovery rate (FDR) modified by the Benjamini–Hochberg method, reflecting the proportion of expected false positives among all essential findings. Lower values suggest a more reliable association between the specific functional term and input genes. FDR: The original rate of false positives before multiple hypothesis testing, representing the proportion of expected false positives among all tests conducted.*

**Table 5:**
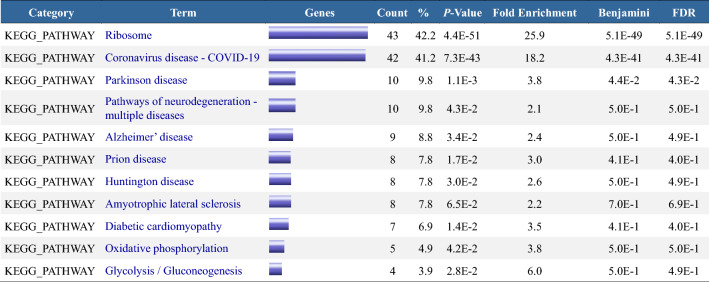
Pathway enrichment analysis (DAVID) of 102 DEGs in group 2.

*KEGG (Kyoto Encyclopedia of Genes and Genomes): The pathway database utilized in this study is aimed at analyzing the enrichment of gene sets within biological pathways. Category: Functional category, indicating the primary classification of genes within the KEGG pathways. Term: Specific functional description elaborating on the role or function of gene sets within particular KEGG pathways. Count: Number of enriched genes, representing the number of genes identified as differentially expressed within the specified functional term.*

*P-value: Statistical significance, used to assess whether the enrichment of a specific functional term is statistically significant, calculated based on the adjusted Fisher's test. Benjamini: False discovery rate (FDR) modified by the Benjamini–Hochberg method, reflecting the proportion of expected false positives within all significant findings. Lower values indicate a more reliable association between the specific functional term and input genes. FDR (false discovery rate): The original rate of false positives before multiple hypothesis testing, representing the proportion of expected false positives among all tests conducted.*

This approach of utilizing advanced bioinformatics tools like DAVID for enrichment analysis and SangerBox for visualization provides a powerful means to interpret and present complex genomic data. The GO and KEGG analyses offer a deeper understanding of the functional and biological significance of the genes implicated in PCOS, revealing their involvement in critical biological processes, pathways, and molecular functions. The visual representation of these analyses facilitates a more precise and intuitive understanding of the data, enabling researchers to communicate their findings and hypotheses more effectively.

### PPI analysis

PPI analysis was conducted using the STRING online tool to explore the intrinsic connections among common differentially expressed genes (co-DEGs)and understand the interrelationships among proteins expressed by these genes. Two sets of 108 and 102 co-DEGs were input into the STRING database, selecting “Multiple sequences” and specifying the biological classification as “Homo sapiens” for protein interaction analysis.

#### Group 1

**Figure 11:**
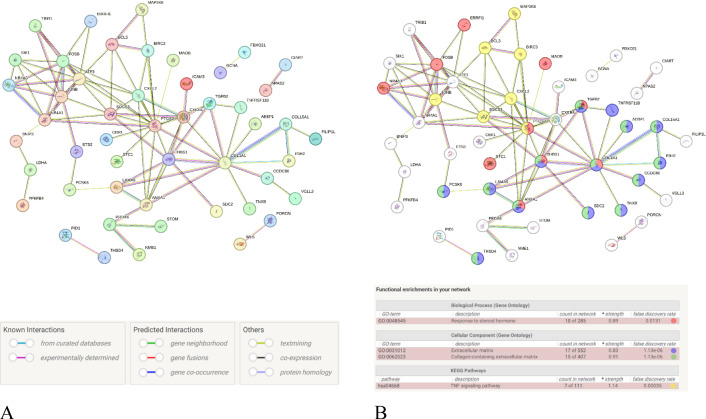
PPI network of the first set of co-DEGs and the labeled PPI network. (**A**), The PPI (protein–protein interaction) Network Diagram. (**B**), The PPI Network identified with high-strength functional and pathway enrichment.

After excluding isolated proteins, the PPI network derived from 108 co-DEGs comprised 89 nodes and 88 interaction edges in Fig. 11. The network exhibited an average node degree of 1.98 and an average local clustering coefficient of 0.385, indicating moderate interconnectivity among the nodes. The PPI enrichment p-value was < 1.0e-16.

Based on node degree data, 15 proteins with connecting nodes ≥ 5 included *PTGS2, COL1A1, ATF3, FOSB, JUNB, CXCR4, NR4A1, SOCS3, THBS1, ANXA1, CXCL2, BCL3, NR4A3, SIK1*, and *TGFB2*. Dark-colored nodes were *PRDX6, ETS2, IFIT3, CYB5A, GAMT, FBXO21, ACSM3, SIK1, SPAG4,* and *JUNB* (for protein functions, see Supplementary Tables 1 and 3). Relevant data in TSV format were exported for subsequent analysis. The resulting functional and pathway enrichment data are presented in Fig. 10B and Supplementary Table 5.

#### Group 2

**Figure 12:**
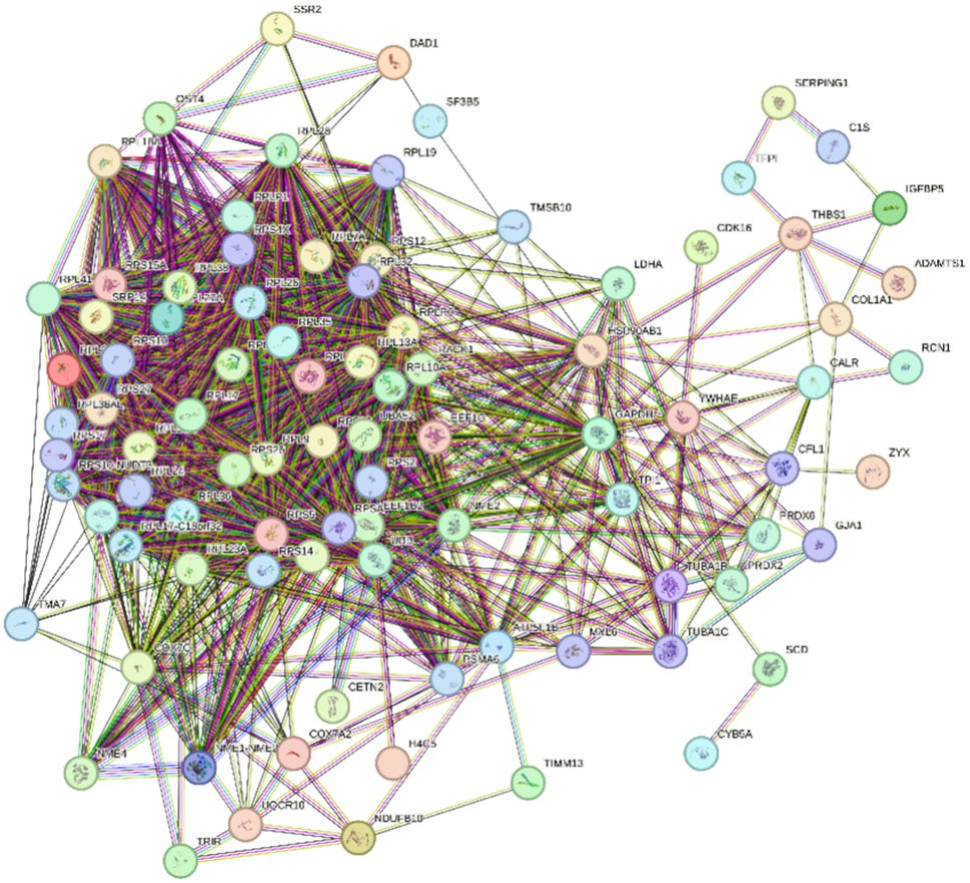
PPI Network of the second set of co-DEGs. Not all have been labeled due to the large number of high-strength functional and pathway enrichments. Relevant content can be found in Supplementary Tables 6 and 7.

The PPI network, derived from 102 co-DEGs and excluding isolated proteins, included 95 nodes and 1,409 edges, with an average local clustering coefficient of 0.695 and an average node degree of 29.7, suggesting a highly interconnected network with a significant scale and evident modular structure (Fig. 12). The PPI enrichment p-value was < 1.0e-16.

Based on node degree data, 55 proteins with connecting nodes ≥ 20 included *RPLP0, RPS2, RPL13A, EEF1G, RPS16, RPSA, RPL9, EEF1B2, RPL23A, RPS14, UBA52, RACK1, RPS11, RPS5, RPL15, RPL17, RPL24, RPL26, RPL30, RPL35, RPL35A, RPL36, RPL38, RPL19, RPL10A, RPL21, RPL27A, RPL32, RPL36AL, RPL7A, RPS12, RPS28, RPS4X, RPL18A, RPL28, RPS15A, RPS17, RPS26, RPS27, RPLP1, RPS10, UBB, RPL17-C18orf32, SRP14, RPS10-NUDT3, GAPDH, NME2, RPL41, HSP90AB1, ATP5F1B, COX7C, OST4, TPI1, PSMA6, NME1-NME2*. Dark-colored nodes included *RPS27, RPL36, SF3B5, NME2, RPS14, RPL30, TFPI, RPL1, RPS11, RPL24, RPL38, TMSB10, RPS5, ZYX, TUBA1C, CETN2*. The functional and pathway enrichment results are presented in Supplementary Tables 6–9. The data in TSV format were exported for further analysis.

### HUB Genes

The TSV file obtained from the previous step was imported into Cytoscape software, and the cytoHubba plugin was employed to analyze HUB genes^14^.

#### Group 1

**Figure 13:**
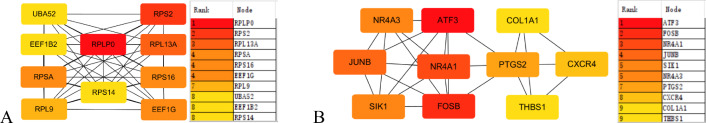
Hub genes identified through analysis with the cytoHubba plugin in Cytoscape software. (**A**), Hub genes of the first group (using the MMC method). (**B**), Hub genes of the second group (using the Degree method).

The PPI network exhibited a modest scale with limited nodes, analyzed using the MMC method. The top 10 HUB genes identified include *ATF3, FOSB, NR4A1, JUNB, SIK1, NR4A3, PTGS2, CXCR4, COL1A1*, and *THBS1* (as shown in Fig. 13A). Consultation of Gencard provided detailed gene names and aliases (see Supplementary Table 8). These genes encompass 17 high-scoring pathways (see Supplementary Table 10) and 21 functions (see Supplementary Table 12). Briefly, *NR4A1, FOSB*, and *JUNB* are linked with transcriptional regulation; *SIK1* is associated with cellular signal transduction; *CXCR4* is related to inflammation and immunity; *COL1A1* is connected to connective tissue and skeletal development. The top 17 pathways include immune inflammation (*IL-1, Toll, TCR, NF-kB*, etc.), signal transduction (*ERK, SMAD*, etc.), and tumor metastasis (*VEGF*), playing critical roles in immune responses, inflammation, cell proliferation, and apoptosis. For instance, the *NF-κB* pathway is central to inflammation and immune responses, while the *ERK* pathway is significant in cellular proliferation and differentiation. The top 21 functions are related to transcriptional regulation, inflammatory responses, embryonic development, and hormonal responses. For example, *NR4A1* is linked to glucocorticoid responses and *JUNB/FOSB* to transcriptional regulation; smooth muscle cell proliferation is associated with vascular remodeling and tumor growth, and cellular responses to corticotropin-releasing hormones are connected to stress and inflammatory responses. HUB genes regulate these processes by participating in them.

#### Group 2

The PPI network with more nodes was analyzed using the Degree method. The top 10 HUB genes identified were *RPLP0, RPS2, RPL13A, RPSA, RPS16, EEF1G, RPL9, UBA52, EEF1B2*, and *RPS14* (as shown in Fig. 13B). Names, gene full names, and aliases were obtained from Gencard (see Supplementary Table 9). These genes are involved in 14 high-scoring pathways (see Supplementary Table 11) and have 18 function attributes (see Supplementary Table 13). These genes mainly encode ribosomal proteins, including the large subunit (*RPLP0, RPL13A, RPL9*), small subunit (*RPS2, RPS16, RPS14*), and auxiliary factors (*EEF1G, EEF1B2, UBA52*). Their functions focus on ribosomal assembly, RNA binding, and translation elongation, playing crucial roles in protein translation.

Pathways include viral infection (SARS-CoV-2), RNA processing, and protein metabolism, consistent with ribosomal translation functions. For instance, SARS-CoV-2 exploits host translation machinery to express viral proteins, where ribosomal HUB genes may play critical roles. These genes are significant in neural system development, cellular responses to stimuli, and metabolism.

These HUB genes encode ribosome components and are regulatory factors in protein synthesis. They participate in normal protein synthesis and act as effectors during viral infections. By regulating the translation process, they affect various physiological and pathological processes such as tumors, inflammation, and viral infections.

*RPLP0, RPS2, RPL13A,* etc., are associated with RNA binding and ribosomes located within the same module; UBA52 is related to the ubiquitination process. They likely collectively regulate intracellular protein synthesis.

## Discussion

The susceptibility to PCOS can be inherited through epigenetic variations of susceptible alleles and developmental programming. Nutritional and other environmental factors also influence the incidence and severity of PCOS^15^.

By integrating multiple datasets and analyzing significantly differentially expressed genes, important discoveries were made in studying genes related to PCOS, systematically revealing for the first time the significant implications of two sets of hub genes as potential biomarkers for PCOS. The “HUB Genes” section will elaborate in detail on the biological functions of these genes and their association with the pathogenesis of PCOS.

### EO2R analysis

Gene-specific expression in different tissues results from differentiation, and a typical expression pattern in various tissues of the same system indicates interconnectivity. For example, the *ER* gene is expressed in both the ovaries and uterus^16^; the proliferation gene *PCNA*^17^ is detectable in the reproductive system; genes involved in cell cycle regulation, apoptosis, differentiation, and signal transduction are expressed in follicle cells and granulosa cells^18^. Their expression across multiple tissues and cells reflects the structural and functional consistency within the system. This study analyzed the DEGs in the ovaries, follicles, and endometrium of PCOS patients from the GEO database, yielding two sets of co-DEGs. The first set comprises 108 common DEGs between granulosa cells GSE155489 and endometrial tissue GSE226146, and the second set consists of 102 co-DEGs between follicular fluid exosomes GSE159466 and endometrial tissue GSE226146.

The presence of co-DEGs in experiments from different sources can be attributed to two main factors:(1) Common Etiology: a. Identical or similar treatment or exposure (disease) leading to a typical gene response. b. DEGs are functionally associated or belong to the same pathway, participating in a shared response. c. Multiple sample groups represent a common physiological or pathological state, leading to consistent changes in key genes. (2) Systemic Bias: a. Using identical reagents or equipment (even the same batch of reagents or the same device) could result in systematic errors. b. Using the same data preprocessing, normalization, and differential analysis processes might falsely identify some genes as differentially expressed. Given that the samples share the same disease state, co-DEGs’ presence primarily reflects the commonalities in the pathophysiological process of PCOS.

GEO2R is a web-based tool that employs R programming to analyze gene expression data from microarray experiments deposited in the GEO database. It identifies differentially expressed genes (DEGs) between different experimental conditions. While GEO2R provides a standardized and straightforward method for initial DEG screening, it has certain limitations. Firstly, GEO2R uses fixed parameters for analysis, whereas researchers conducting analysis directly in R can choose different parameters and packages, such as limma, EdgeR, and Deseq2 function packages, potentially leading to varying results. Secondly, GEO2R cannot eliminate batch effects, which are systematic biases that may exist between different batches of data. Additional methods, such as Venn diagram analysis, should be employed to mitigate the influence of batch effects, as in the present study. Despite these limitations, GEO2R remains a valuable tool for preliminary identification of DEGs. In our research, GEO2R analysis facilitated the detection of PCOS-associated DEGs, laying the groundwork for subsequent bioinformatic analyses.

### DGEs analysis

#### Parameter settings

(1) Selection Criteria: A *Padj* (adjusted* P* value after multiple testing correction) < 0.05 is statistically significant and non-random. |log2FC|≥ 1, or equivalently |FC|≥ 2, measures the extent of gene expression change (FC: fold change), and at this threshold, the difference in gene expression is considered biologically significant. Meeting both *P*adj < 0.05 and |log2FC|≥ 1 criterion indicates that gene expression differences are significant both statistically and biologically.(2) Enrichment Conditions: EASE is a commonly used index for gene enrichment analysis. An empirical setting of 0.1 ensures the reliability and interpretability of the results, providing a sufficient number of enrichment outcomes while filtering out non-specific background enrichment.

#### Advantages of the bubble chart

(1)Efficiently displays three-dimensional information in a high-density format. (2) Utilizes size, color, and position to highlight the importance of DEGs' functions/pathways. (3) Offers an intuitive visualization of the expression patterns of DEGs.

#### Dual enrichment analysis

The co-DEGs are analyzed for functional and pathway enrichment using DAVID and STRING online tools. Each tool offers different coverage of databases, algorithms, visualization capabilities, and extended functionalities. STRING provides more comprehensive features and a higher degree of visualization, while DAVID covers more authoritative databases. Together, they complement each other to yield a more comprehensive and reliable analysis outcome.

### Enrichment analysis

The functional and pathway enrichment analysis of co-DEGs provides insight into the pathophysiological processes of diseases, revealing potential biomarkers and therapeutic targets^19^.

#### Group 1

##### Results interpretation

(1) Function: Mainly involves regulating extracellular matrix composition and cell cytoskeleton connections, affecting cell survival processes, which includes enrichment in biological processes related to a. extracellular matrix structural constituent, extracellular space, extracellular region, extracellular exosome, endoplasmic reticulum lumen, and endoplasmic reticulum, indicating abnormalities in cellular matrix synthesis; b. cell–cell adhesion and cell surface suggest that intercellular adhesion is affected; c. negative regulation of cell proliferation and negative regulation of the apoptotic process indicate abnormal cell survival states. (2) Pathway: The TNF signaling pathway is related to cell proliferation, differentiation, apoptosis, and inflammatory response^20,21^. The osteoclast differentiation pathway is related to extracellular matrix metabolism and skeletal development. Amoebiasis, Malaria, and Human papillomavirus infection pathways are related to the immune response to pathogen infections. Inflammation and immune-related pathways like IL-17 signaling and Proteoglycans in cancer are also enriched. Overall, pathways including TNF, IL-17 inflammatory factor signaling, bone development pathways, and host immune response to pathogens are enriched, participating in response to inflammatory factors and stress stimuli by regulating the synthesis and secretion of extracellular matrix components, changes in the bone microenvironment, and cell survival state.

##### Bubble chart

(1) Functiont: In the upper left corner, genes related to extracellular matrix structural components, response to exogenous stimuli, and functions related to extracellular matrix and extracellular space are not numerous but have the smallest FDR and *P* values, indicating significance. The giant bubble “protein binding” contains the most genes with higher FDR and *P* values, indicating weaker statistical significance. “Cytoplasm” is the second-largest gene number and has lower FDR and *P* values, signifying its relevance. (2) Pathway : The “TNF signaling pathway” contains the most genes, with the highest FDR and smallest *P* values, indicating its significant role. It plays a crucial role in many biological processes, including cell growth, differentiation, and apoptosis, as well as in diseases like cancer and autoimmune diseases. ECM-receptor interaction pathway has many highly significant genes, suggesting changes in cell adhesion activity, regular binding, and signaling between cells and the matrix, affecting normal cell survival, movement, proliferation, etc^22,23^.

#### Group 2

##### Results interpretation

(1) Function: Mainly involves ribosome and protein translation processes. Functions related to cytoplasmic ribosomes, such as “cytoplasmic translation” and “ribosome,” reflect active ribosome function and protein translation processes, potentially associated with proliferative diseases like tumors, reflecting a high proliferation state of the tissue. “Focal adhesion” enrichment suggests potential impacts on cell adhesion functions related to tumor metastasis and adhesion. “Extracellular exosome” enrichment indicates a relation to cell secretory vesicles, requiring attention to changes in cell secretion and microenvironment. These datasets relate enhanced proliferation and protein synthesis functions with other active cellular activities. Terms like “RNA binding” and “protein binding” are also enriched but with general statistical significance. Functional enrichment reveals changes in biological processes related to ribosomes, highlighting the importance of ribosome-related functions. (2) Pathway : The ribosome pathway is highly significant, with the most genes and smallest* P* values, indicating differences in protein synthesis processes among samples. Coronavirus infection-related pathways are also highly enriched, with the second-highest gene count and *P* values, indicating abnormal viral infection or immune responses in samples. Parkinson’s disease and other neurological system pathways show mild enrichment, suggesting changes in the nervous system. Glycolysis/Insulin signaling and different metabolism-related pathways are also mildly enriched. Overall, pathways are mainly enriched in ribosomes and immune responses.

##### Bubble chart

**(**1) Function: “Protein binding,” “cytosol,” and “cytoplasm” contain the most genes, indicating co-DEGs primarily function in the cytoplasm, involved in processes like protein synthesis, folding, and transport. “Protein binding” means binding of proteins to other molecules (proteins, RNA, DNA, small molecules) and is fundamental to many biological processes (signal transduction, metabolism, cell division). “Cytosol” and “cytoplasm” contain numerous proteins, RNA, DNA, small molecules, etc., and are sites for many biological processes. These three terms’ significant functional enrichment suggests phenomena affecting intracellular space structure/function, biological processes in the cytoplasm, and protein binding. Terms like “cytoplasmic translation,” “cytosolic ribosome,” “structural constituent of ribosome,” “translation,” etc., are enriched, indicating DEGs play a crucial role in protein synthesis and translation. “Negative regulation of translation” enrichment suggests regulation of protein translation. “Endoplasmic reticulum,” “nucleolus,” “vesicle,” “RNA binding,” and “ribonucleoprotein complex” enrichment indicate co-DEGs play essential roles in ribosome biosynthesis, RNA processing and transport, protein translation, and secretion. “Focal adhesion,” “extracellular exosome,” “membrane,” and “synapse” enrichment show DEGs play significant roles in intercellular communication, signal transduction, and cell adhesion. These genes are essential in cellular processes like protein synthesis, translation, transport, and signal transduction. (2) Pathway : Giant bubbles are “Ribosome” and “Coronavirus disease—COVID-19”, with the smallest FDR and *P* values. “Ribosome” is a complex required for protein synthesis, composed of rRNA and proteins, and is involved in all protein synthesis, indicating that this group of DEGs mainly affects the biological mechanisms of protein synthesis. “Coronavirus disease—COVID-19” refers to the disease caused by the SARS-CoV-2 virus. SARS-CoV-2 infects cellular organelles, including mitochondria and ribosomes. This pathway's enrichment indicates biological mechanisms affecting the virus's infection. The remaining pathways have larger FDR and *P* values and fewer related genes, which are less significant than the first two. For example, “Diabetic cardiomyopathy” relates to diabetes-associated heart disease. “Glycolysis / Gluconeogenesis” are the two main pathways of carbohydrate metabolism in cells; “Oxidative phosphorylation” is the main pathway for energy production in cells; the rest are related to neurodegenerative diseases.

### PPI analysis

#### Group 1

Each protein, on average, interacts with 1.98 other proteins. A lower clustering coefficient suggests that the proteins are not very tightly connected. The clustering in the PPI network is significantly enriched, indicating biologically meaningful interactions. Directed interactions and regulatory relationships among proteins may be critical regulators in specific pathways or processes.

- Proteins with connecting nodes ≥ 5: There are 15 such proteins whose functions are detailed in Supplementary Table 1. These proteins are closely related to immune-inflammatory responses, tissue damage, and repair processes. Specifically, they regulate inflammation, gene expression, immune cell function, connective tissue and extracellular matrix metabolism, cell proliferation and differentiation, and intracellular metabolism.
- Functions of 10 dark-colored node proteins: Refer to Supplementary Table 3. These crucial proteins involve various processes, such as antioxidative stress, metabolic regulation, and cell cycle control. Abnormalities in these proteins can lead to oxidative damage, metabolic disorders, and cell cycle dysregulation. For example, *PRDX6* (Peroxiredoxin) is an antioxidant protein that reduces hydrogen peroxide and lipid peroxides, protecting cells from oxidative damage. It is also involved in phosphatidylcholine synthesis. *ETS2*, a transcription factor, activates the transcription of various genes that regulate cell proliferation and differentiation. *IFIT3* is an antiviral protein that inhibits viral replication and enhances antiviral responses. *CYB5A* (Cytochrome b5) participates in electron transfer, supporting redox reactions. *GAMT* is involved in the creatine synthesis pathway, crucial for nervous system development. *FBXO21* may act as part of a ubiquitin ligase complex, which regulates protein ubiquitination and degradation. *SIK1*, a serine/threonine kinase, regulates the cell cycle and gluconeogenesis and acts as a tumor suppressor; SPAG4 and JUNB regulate cell proliferation and differentiation.
- Functional and Pathway Enrichment (Supplementary Table 5): “Response to steroid hormone” enrichment suggests co-DEGs play roles in response to steroid hormones. Cellular component enrichment in “Extracellular matrix” and “Collagen-containing extracellular matrix” indicates that co-DEGs are related to the structure and function of the extracellular matrix, especially collagen-containing matrices. “TNF signaling pathway” enrichment suggests co-DEGs are involved in TNF signaling, potentially related to inflammatory and immune responses. These enrichment results are meaningful for understanding PCOS's onset, progression, and treatment.

#### Group 2

The average node degree is 29.7. A high average local clustering coefficient (near 0.7) suggests tight connections. The clustering in the PPI network is highly significant, meaning biologically meaningful connections. The proteins are involved in complex and close interactions, participating in critical biological processes or signaling pathways, and are central components of critical pathways or regulatory networks.

- Connecting nodes ≥ 20: The 55 proteins are detailed in Supplementary Table 2. These proteins are involved in fundamental cellular activities like gene expression, protein synthesis, metabolism, protein folding, degradation, and mitochondrial functions. They include ribosomal proteins (e.g., *RPL, RPS*) which are part of the ribosome and responsible for protein synthesis, proteins involved in protein synthesis (e.g., *EEF1B2, EEF1G*), enzymes involved in cellular metabolism (e.g., *GAPDH, TPI1, NME1/2*), molecular chaperones like *HSP90AB1*, components of mitochondrial respiratory chain complexes (e.g., *COX7C, ATP5F1B*) or protein degradation complexes (e.g., *PSMA6*), and fusion proteins (e.g., *RPL17-C18orf32, NME1-NME2, RPS10-NUDT3*).
- Functions of 16 dark-colored node proteins: Refer to Supplementary Table 4. They are related to nucleotide metabolism, protein production, cytoskeletal processes, etc. Abnormalities in these proteins could lead to metabolic disorders, coagulation dysfunctions, and cell motility disorders. Ribosomal proteins (e.g., *RPS27*, *RPL36, RPL17, RPS11, RPL24, RPL38*) are involved in ribosome assembly, post-transcriptional modifications of mRNA, and protein translation. *SF3B5*, a part of the spliceosome, is involved in pre-mRNA splicing. *NME2* is involved in nucleotide synthesis and regulates Rho signaling and *MYC* gene expression. RPS14 is a structural component of the ribosome. *TFPI* (Tissue Factor Pathway Inhibitor) directly inhibits coagulation factor X (Xa) in coagulation regulation. *TMSB10* is involved in cytoskeletal reorganization. *ZYX*, an adhesion protein, mediates gene expression changes triggered by adhesion stimuli. *TUBA1C*, a major component of microtubules, and *CETN2* are involved in the structure and function of the microtubule organizing center.
- Functional and Pathway Enrichment (Supplementary Table 6): Biological processes like “Cytoplasmic translation,” “Ribosomal small subunit assembly,” “Ribosomal large subunit assembly,” “Ribosome assembly,” “Translation,” etc., indicate co-DGEs play significant roles in protein synthesis and metabolism. Other enriched biological processes are related to nucleotide biosynthesis and metabolism, such as “UTP biosynthetic process,” “GTP biosynthetic process,” “CTP biosynthetic process,” “Nucleoside diphosphate phosphorylation,” “Purine ribonucleoside triphosphate biosynthesis,” etc.

The molecular function enrichment reflects vigorous tissue metabolism and growth, with active gene expression regulation, protein synthesis, structural formation processes, and intercellular adhesion interactions. For example, enrichment in the structural constituent of the ribosome and rRNA binding indicates enhanced protein translation and ribosome assembly activities. Structural molecule activity enrichment suggests increased activities in forming cellular structures and maintaining cell morphology.

Cell component enrichment includes a. “Ribosomal subunit,” “Polysome,” “Large ribosomal subunit,” “Cytosolic small ribosomal subunit,” etc., indicating the network's involvement in protein synthesis and processing. b. “Focal adhesion” suggests participation in transporting proteins, lipids, and other molecules. Overall, cell component enrichment results relate to protein synthesis. “GAIT complex” has a high strength (2.01), implying biological significance, and a very low FDR value, indicating its statistical significance. Entries such as “Cytosolic ribosome” and “Large ribosomal subunit” have a relatively high number of genes and very low FDR values, suggesting these genes may be essential in this network.

KEGG pathways (Supplementary Table 7): a. The “Ribosome” pathway involves 39 genes, with an intensity of 1.79 and a very low FDR (6.73e-54), indicating its extreme importance. The ribosome, as the site of protein synthesis, in conjunction with other pathways, may be related to neuronal repair and regeneration. b. The “Oxidative phosphorylation” pathway includes five genes, with an intensity of 0.9 and an FDR of 0.0228, highlighting its importance. It is the main pathway for cellular energy production, and its enrichment suggests an upregulation of mitochondrial function, potentially as a protective mechanism against damage. c. Pathways related to neurodegenerative diseases such as “Parkinson’s disease,” “Prion disease,” “Huntington’s disease,” “Alzheimer’s disease,” and “Amyotrophic lateral sclerosis,” although of lower intensity, have low FDR values, indicating their significance.

In conclusion, the pathway enrichment of this group of co-DEGs involves protein synthesis, energy metabolism, and pathways related to neurodegenerative diseases. The enrichment of biological processes related to endoplasmic reticulum protein synthesis aligns with environmental factors implicated in the pathogenesis of PCOS, such as follicular endoplasmic reticulum stress (ER stress) and intrauterine hyperandrogenism.

### HUB genes

#### Group 1

This group of HUB genes is crucial in regulating inflammatory responses and the extracellular matrix. Functional enrichment includes cytokine regulation, inflammatory responses, and extracellular matrix organization. Details are as follows:

*ATF3* shows an upregulation trend in PCOS cystic embryos^24^, playing a vital role in cellular stress and apoptosis, which may have profound implications for maintaining embryo homeostasis and developmental processes. However, the expression of *ATF3* is downregulated in uterine leiomyomas^25–30^, indicating different regulatory patterns under various pathological conditions. Similarly, a study found a significant reduction in the expression of *FOSB* in the adipose tissue of PCOS patients^31^, which is also upregulated in this study.

Members of the NR4A family, including *NR4A1, NR4A2,* and *NR4A3*, play complex roles in the pathophysiology of PCOS. *NR4A1*, one of the differentially expressed genes in PCOS^32^, is regulated by androgens^33^, while its transcriptional activity is inhibited by dietary medium-chain fatty acids^34^. Additionally, *NR4A3* is crucial in early mouse embryogenesis^35^ and shows differential expression under follicular hypoxic conditions^36^, potentially leading to ovarian follicle aging and a decline in oocyte quality. Low-frequency electrical stimulation, an emerging treatment method^37^, can regulate epigenetic and gene transcription levels in adipose tissue in a rat model of PCOS, including *FOSB, JUNB, NR4A3,* and *NR4A2*^37,38^. Note: Proteins encoded by *NR4A2, NR4A1*, and *NR4A3* are highly homologous in structure^32^.

*JUNB* regulates various cell types and physiological processes. For instance, it plays a vital role in the epigenetic changes and differentiation of hematopoietic stem cells^39^ and promotes lesion invasion and metastasis in endometriosis^40^. *FOS, JUNB,* and *JUND* expression in human ovulatory follicles is significantly increased following treatment with human chorionic gonadotropin^41^. JUNB is involved in the proliferation and survival of embryonic tail bud cells^42^, and its overexpression in placental mesenchymal stromal cells (PDMSCs) in pre-eclampsia may affect the regulation of cyclin D1^43^.

*SIK* family members are vital in maintaining cellular energy balance and responding to nutritional deficiency^44,45^. Their expression and function are essential in ovarian function and female fertility, particularly in regulating StAR expression and carbohydrate and lipid metabolism, participating in PCOS-related androgen overproduction^46–48^. SIK inhibitors significantly enhance FSH actions in patients’ granulosa cells^49^.

The expression of *COX-2 (PTGS2)* is increased in a testosterone-induced mouse model of PCOS, indicating its potential role in the pathogenesis of PCOS^50^. Although some studies observed decreased *COX-2* expression in the endometrium^51^ and granulosa cells^52^ of PCOS models, most research supports the overactivation of *COX-2* in PCOS patients, especially in ovarian tissue^53–55^, Which is presumed to be related to hyperandrogenism, increased inflammatory responses, and abnormal follicular development in PCOS patients. Metformin and *p38MAPK* inhibitors can reverse testosterone-induced stress and *COX-2* expression^56,57^, with other potential inhibitors, including peppermint extract^58^ and high doses of EPA^59^. In PCOS models with reduced *COX-2* expression, the regulatory role of vitamin D may improve the effects of PCOS on the lower limb veins^60^ and aortic endothelial cells^61^.

*CXCR4*, a G-protein-coupled receptor, plays a regulatory role in the pathogenesis of PCOS, affecting immune responses, cell migration, and inflammation through its interaction with *CXCL12*^62,63^. In a rat model of PCOS, the expression of *CXCL12, CXCR4,* and *CXCR7* in the ovaries is reduced, while apoptosis in granulosa cells (GC) is increased^64^. The overexpression of *ECM* is associated with the enlargement of uterine fibroids^25^. It plays roles in cell morphology, proliferation, and differentiation and is a reservoir for growth factors and cytokines, mediating their activation and turnover. Uterine fibroids, the most common benign tumors of the female reproductive system, can cause various clinical symptoms, including infertility, miscarriage, premature birth, and placental abruption.

#### Group 2

The HUB gene package includes *RPLP0, RPL13A,* and *RPL9*, which belong to the large subunit family of ribosomal proteins, and *RPS2, RPSA, RPS16, RPS14*, belonging to the small subunit family. *EEF1G* and *EEF1B2* encode subunits of translation elongation factor 1, playing a core role in the translation elongation step of protein synthesis^65^ and are significantly associated with tumor development^66–68^. These genes are involved in protein synthesis, and their aberrant expression could lead to improper synthesis of hormones or hormone-signaling related proteins, thereby broadly affecting endocrine and metabolic functions and triggering clinical symptoms of PCOS, such as ovarian hormonal imbalance and insulin resistance.

*RPLP0* is proven to be a gene with extremely high expression stability in human ovarian tissue cells and follicles^69^, a finding also validated in mouse models^70^. Therefore, *RPLP0* can serve as a reference gene for analysis^71^, used to monitor the normalization of target gene expression^72^. The *RPS2* gene has been identified as a HUB gene in conditions including premature ovarian insufficiency with infertility^73^ and unexplained recurrent spontaneous abortion^74^. In the Culex pipiens mosquito, inhibition of *RPS2* arrested ovarian development (diapause), and its expression shutdown could be potentially valuable for improving diapause in adult female mosquitoes^75^.

The protein encoded by *RPL13A* plays a vital role in inflammatory responses, embryonic development, and oocyte maturation and is essential for the completion of preimplantation embryo development in mice^76^. Due to its superior performance, *RPL13A* has become the preferred gene for qPCR studies of mesenchymal stromal cells (MSCs) derived from bone marrow and placenta^77^. The *RPSA* gene is associated with congenital developmental defects, such as asplenia, in humans^78^ and Xenopus^79^. In Alzheimer’s patients’ cerebral vasculature, an upregulation of 28 ribosomal proteins, including *RPLP0, RPSA, RPS16, RPS14,* and *RPS2*^80^. In high-grade gliomas, CD8^+^ cell infiltration markers include *RPS16*^81^. *RPL9, RPLP0, RPS15, UBA52,* and *RPL13* have also been identified as HUB genes in male infertility^82^. *UbA52* encodes a ubiquitin ribosomal fusion protein, whose downregulation affects ubiquitin formation and oocyte development^83^ and is indispensable in early embryogenesis^84^. Additionally, it is a stably expressed gene in bovine ovaries^85^, involved in early pregnancy changes in the porcine endometrium, and can be induced by interferons^86^. *UbA52*-deficient mouse models die during embryogenesis, exhibiting reduced protein synthesis and cell cycle arrest^87^. In mice, *UbA52* is also considered a gene responsive to high glucose stimuli and is involved in developing diabetic nephropathy^88^.

### The issue of upregulation and downregulation

DEGs are associated with PCOS, potentially as causes or as secondary changes during the disease course. Some of these genes exhibit contrasting up/downregulation changes. For instance, *HMGA2* is highly expressed in the granulosa cells (GCs) of women with PCOS^89,90^, increasing GC cell vitality and proliferation while reducing apoptosis, which seems contradictory to the pathophysiological mechanisms of PCOS. A plausible explanation might be that overexpression of *HMGA2* merely reflects abnormalities in cell differentiation and maturation; alternatively, the abnormal proliferation might be a phenotype in the course of PCOS, not a direct cause of infertility. Instead, it could be participating in the onset and progression of PCOS in conjunction with other factors.

## Conclusions and prospects

The present study provides novel insights into the underlying mechanisms of pathological changes in PCOS by analyzing co-DEGs. It enhances our understanding of PCOS’s cellular biological state and regulatory mechanisms, offering valuable clues for disease research. Existing evidence indicates a close relationship between PCOS onset and interactions of multiple genetic and environmental factors, with a higher familial aggregation than the general population^91–93^, which strongly implicates the involvement of genetic factors. These factors are widely believed to likely involve genes regulating ovarian and adrenal hormone synthesis and insulin-related genes^94^.

The HUB genes identified in this study include (1) Genes encoding transcription factors and genes encoding cytokines, which play a central role in inflammation responses and extracellular matrix regulation; (2) Genes encoding ribosomal protein, which influence the synthesis of hormone-related proteins, thereby involving the cell’s endocrine and metabolic processes. The enrichment of co-DEGs’ functions and pathways mainly focuses on two aspects: (1) Regulating the extracellular matrix and the cytoskeletal structure, which not only affects intercellular connections and survival states but also participates in signal transduction; (2) Involving ribosome composition and protein translation processes, as well as aspects of viral response, neural metabolism, and cellular energy production.

These genes and their associated functions and pathways provide a new perspective for understanding DEGs’ role in PCOS, informing future intervention strategies. For instance, the enrichment of genes related to protein translation and intracellular transport suggests that abnormal cell growth and proliferation in PCOS might play a vital role in the pathological process, and intervention targeting this could be a new approach. Furthermore, functional enrichment analysis also revealed shared pathological processes between PCOS and various diseases such as cancer, neurological diseases, and autoimmune diseases. These processes are associated with cytoskeletal organization, glycolysis, and heat shock response, indicating the potential to uncover the underlying causes of PCOS and develop new therapeutic approaches.

Elucidating the pathogenesis of PCOS also requires considering gene-environment interactions^94^. The environmentally induced epigenetic heritability can produce transgenerational effects^92,95^, which may have a more significant impact on the pathological development of PCOS than purely genetic factors^91^. Therefore, future treatment strategies should adopt an interdisciplinary approach, implemented by a team comprising experts in nutrition, endocrinology, obstetrics, gynecology, and reproductive medicine^94,96^. For example, nutritional interventions (supplementation with vitamin D, B vitamins, and *ω*-3 fatty acids) can improve PCOS symptoms by regulating metabolism and hormone levels and may resolve infertility issues^15^.

## Limitations

This study included samples from different tissues, such as granulosa cells from ovarian follicles, endometrial tissue, and follicular fluid exosomes, providing an opportunity to study PCOS’s pathological mechanisms and related molecular changes comprehensively. However, due to current limitations in resources, funding, time, and the scope of the article, we are unable to perform experimental validation at this time. We recognize that experimental validation would significantly strengthen our results, especially considering potential differences in gene expression and molecular mechanisms among various reproductive tissues. In the future, we plan to prioritize experimental validation.

Additionally, the study involved a relatively small number of microarray datasets, including only three groups with 20 samples, primarily from Tianjin, Shandong Province, and Jiangsu Province in China. Although the prevalence of PCOS does not significantly differ among ethnic groups, there may be phenotypic variations^91^. Therefore, future research should expand the sample size and include a more diverse population to validate the results of this study. Furthermore, integrating multi-omics data, such as proteomics, metabolomics, and microbiome data, will help to more comprehensively understand the molecular mechanisms of PCOS.

### Supplementary Information

Supplementary Tables.

## Data Availability

The datasets generated and analyzed during the current study are available in the Mendeley repository (https://data.mendeley.com/datasets/3n3xz759hb/1) and from the corresponding author upon reasonable request.
