# Supplementary material for: Transcriptome profiling reveals dysregulation of inflammatory and protein synthesis genes in PCOS
Source: Sci Rep. 2024 Jul 18;14:16596. doi: 10.1038/s41598-024-67461-4 (PMC11258128; doi:10.1038/s41598-024-67461-4)
Supplement: Supplementary file 1 — Supplementary Tables. [file 41598_2024_67461_MOESM1_ESM.docx]

**Transcriptome Profiling Reveals Dysregulation of Inflammatory**

**and Protein Synthesis Genes in PCOS**

## **SUPPLEMENTARY**

**Supplementary Table 1：Annotations (GeneCards) of 15 proteins with node degree >5 in the first group of PPI networks**

| **Gene** | **Annotations** |
| --- | --- |
| PTGS2 | PTGS2 encodes the cyclooxygenase COX-2 enzyme, which plays an important role in prostaglandin biosynthesis and is expressed in cells in response to pro-inflammatory stimuli. |
| COL1A1 | COL1A1 encodes the pro-alpha1 chains of type I collagen, the major component of collagen fibrils found in connective tissues. |
| ATF3 | ATF3 is a transcriptional regulator that is activated by various signals including cytokines, genotoxic agents, and stress. It can activate or repress target gene transcription. |
| FOSB | FOSB dimerizes with Jun proteins to form the AP-1 transcription factor complex, which regulates genes involved in cell proliferation and differentiation. |
| JUNB | JUNB is a transcriptional regulator that interacts with Fos proteins to form heterodimeric AP-1 complexes that bind to DNA and regulate gene expression. It has a role in stress response. |
| CXCR4 | CXCR4 is a chemokine receptor that binds CXCL12 and plays a role in immunoregulatory and inflammatory processes as well as in cancer metastasis. |
| NR4A1 | NR4A1 encodes an orphan nuclear receptor that regulates genes involved in metabolism, inflammation, cell proliferation and apoptosis. |
| SOCS3 | SOCS3 regulates cytokine signaling via inhibition of the JAK/STAT pathway and thus acts in a negative feedback loop to restrain inflammatory responses. |
| THBS1 | THBS1 encodes an adhesive glycoprotein that mediates cell-to-cell and cell-to-matrix interactions and plays roles in platelet aggregation, angiogenesis, and tumorigenesis. |
| ANXA1 | ANXA1 has anti-inflammatory activity through inhibiting phospholipase A2 and production of inflammatory mediators, and suppression of neutrophil migration. |
| CXCL2 | CXCL2 is a chemokine that attracts neutrophils and basophils. It is involved in inflammatory responses through regulating leukocyte migration. |
| BCL3 | BCL3 encodes a transcriptional coactivator that interacts with NF-kappa-B homodimers to enhance target gene transcription. It is involved in cell proliferation, differentiation and survival. |
| NR4A3 | NR4A3 is a nuclear receptor that acts as a transcription factor and regulates genes involved in cell growth, apoptosis, inflammation and glucose metabolism. |
| SIK1 | SIK1 is a protein kinase that regulates multiple cellular processes including glucose and lipid metabolism, macrophage polarization and sodium transport. |
| TGFB2 | TGFB2 encodes a secreted ligand of the TGF-beta superfamily. It regulates proliferation, differentiation, adhesion, migration and apoptosis and plays a role in development and immune function. |

**Supplementary Table 2：Annotations of 55 proteins with node degree >20 in the second group of PPI networks (GeneCards)**

| **Gene** | **Annotations** |
| --- | --- |
| RPLP0 | RPLP0 encodes a ribosomal protein that is a component of the 60S subunit. It belongs to the L10P family of ribosomal proteins. It is located in the cytoplasm. |
| RPS2 | RPS2 encodes a ribosomal protein that is a component of the 40S subunit. It belongs to the S2P family of ribosomal proteins. It is located in the cytoplasm. |
| RPL13A | RPL13A encodes a ribosomal protein that is a component of the 60S subunit. It is located in the cytoplasm. It belongs to the L13P family of ribosomal proteins. It may play a role in macular degeneration. |
| EEF1G | EEF1G encodes a subunit of the elongation factor-1 complex, which is responsible for the enzymatic delivery of aminoacyl tRNAs to the ribosome. It is located in the cytoplasm. |
| RPS16 | RPS16 encodes a ribosomal protein that is a component of the 40S subunit. It belongs to the S9P family of ribosomal proteins. It is located in the cytoplasm and nucleolus. |
| RPSA | RPSA encodes a ribosomal protein that is a component of the 40S subunit. It is located in the cytoplasm. It belongs to the S2P family of ribosomal proteins. It binds laminin and may function outside the ribosome. |
| RPL9 | RPL9 encodes a ribosomal protein that is a component of the 60S subunit. It belongs to the L6P family of ribosomal proteins. It is located in the cytoplasm. |
| EEF1B2 | EEF1B2 encodes a subunit of the elongation factor-1 complex, which is responsible for the enzymatic delivery of aminoacyl tRNAs to the ribosome. It is located in the cytoplasm. |
| RPL23A | RPL23A encodes a ribosomal protein that is a component of the 60S subunit. It belongs to the L14P family of ribosomal proteins. It is located in the cytoplasm. |
| RPS14 | RPS14 encodes a ribosomal protein that is a component of the 40S subunit. It belongs to the S11P family of ribosomal proteins. It is located in the cytoplasm. |
| UBA52 | UBA52 encodes a fusion protein of ubiquitin and ribosomal protein L40. Ubiquitin may be involved in protein degradation while L40 is a component of the ribosomal 60S subunit. The encoded protein is located in the cytoplasm. |
| RACK1 | RACK1 encodes a scaffolding protein that interacts with many proteins to regulate various signaling pathways. It is a component of the 40S ribosomal subunit and is located in the cytoplasm. |
| RPS11 | RPS11 encodes a ribosomal protein that is a component of the 40S subunit. It belongs to the S15P family of ribosomal proteins. It is located in the cytoplasm. |
| RPS5 | RPS5 encodes a ribosomal protein that is a component of the 40S subunit. It belongs to the S2P family of ribosomal proteins. It is located in the cytoplasm. |
| RPL15 | RPL15 encodes a ribosomal protein that is a component of the 60S subunit. It belongs to the L15P family of ribosomal proteins. It is located in the cytoplasm. |
| RPL17 | RPL17 encodes a ribosomal protein that is a component of the 60S subunit. It belongs to the L22P family of ribosomal proteins. It is located in the cytoplasm. |
| RPL24 | RPL24 encodes a ribosomal protein that is a component of the 60S subunit. It belongs to the L24P family of ribosomal proteins. It is located in the cytoplasm. |
| RPL26 | RPL26 encodes a ribosomal protein that is a component of the 60S subunit. It belongs to the L24P family of ribosomal proteins. It is located in the cytoplasm. |
| RPL30 | RPL30 encodes a ribosomal protein that is a component of the 60S subunit. It belongs to the L7AE family of ribosomal proteins. It is located in the cytoplasm. |
| RPL35 | RPL35 encodes a ribosomal protein that is a component of the 60S subunit. It belongs to the L29P family of ribosomal proteins. It is located in the cytoplasm. |
| RPL35A | RPL35A encodes a ribosomal protein that is a component of the 60S subunit. It belongs to the L29P family of ribosomal proteins. It is located in the cytoplasm. |
| RPL36 | RPL36 encodes a ribosomal protein that is a component of the 60S subunit. It belongs to the L31e family of ribosomal proteins. It is located in the cytoplasm. |
| RPL38 | RPL38 encodes a ribosomal protein that is a component of the 60S subunit. It belongs to the L38e family of ribosomal proteins. It is located in the cytoplasm. |
| RPL19 | RPL19 encodes a ribosomal protein that is a component of the 60S subunit. It belongs to the L19e family of ribosomal proteins. It is located in the cytoplasm. |
| RPL10A | RPL10A encodes a ribosomal protein that is a component of the 60S subunit. It belongs to the L10e family of ribosomal proteins. It is located in the cytoplasm. |
| RPL21 | RPL21 encodes a ribosomal protein that is a component of the 60S subunit. It belongs to the L21e family of ribosomal proteins. It is located in the cytoplasm. |
| RPL27A | RPL27A encodes a ribosomal protein that is a component of the 60S subunit. It belongs to the L27Ae family of ribosomal proteins. It may be involved in growth regulation. It is located in the cytoplasm. |
| RPL32 | RPL32 encodes a ribosomal protein that is a component of the 60S subunit. It belongs to the L29 family of ribosomal proteins. It is located in the cytoplasm. It may be involved in development of multiple myeloma. |
| RPL36AL | RPL36AL encodes a ribosomal protein that is a component of the 60S subunit. It is a homolog of RPL36A. It is located in the cytoplasm. |
| RPL7A | RPL7A encodes a ribosomal protein that is a component of the 60S subunit. It belongs to the L30P family of ribosomal proteins. It may play a role in coordinating ribosome assembly. It is located in the cytoplasm. |
| RPS12 | RPS12 encodes a ribosomal protein that is a component of the 40S subunit. It belongs to the S12E family of ribosomal proteins. It is located in the cytoplasm. |
| RPS28 | RPS28 encodes a ribosomal protein that is a component of the 40S subunit. It belongs to the S28E family of ribosomal proteins. It is located in the cytoplasm. |
| RPS4X | RPS4X encodes a ribosomal protein that is a component of the 40S subunit. It belongs to the S9E family of ribosomal proteins. It is located in the cytoplasm. This gene is found on the X chromosome. |
| RPL18A | RPL18A encodes a ribosomal protein that is a component of the 60S subunit. It belongs to the L18P family of ribosomal proteins. It is located in the cytoplasm. |
| RPL28 | RPL28 encodes a ribosomal protein that is a component of the 60S subunit. It belongs to the L24P family of ribosomal proteins. It is located in the cytoplasm. |
| RPS15A | RPS15A encodes a ribosomal protein that is a component of the 40S subunit. It belongs to the S19E family of ribosomal proteins. It is located in the cytoplasm. |
| RPS17 | RPS17 encodes a ribosomal protein that is a component of the 40S subunit. It belongs to the S17E family of ribosomal proteins. It is located in the cytoplasm. |
| RPS26 | RPS26 encodes a ribosomal protein that is a component of the 40S subunit. It belongs to the S26E family of ribosomal proteins. It is located in the cytoplasm. |
| RPS27 | RPS27 encodes a ribosomal protein that is a component of the 40S subunit. It belongs to the S27E family of ribosomal proteins. It may bind RNA and play a role in apoptosis. It is located in the cytoplasm. |
| RPLP1 | RPLP1 encodes a ribosomal protein that is a component of the 60S subunit. It belongs to the P1/P2 family of ribosomal proteins. It is located in the cytoplasm. |
| RPS10 | RPS10 encodes a ribosomal protein that is a component of the 40S subunit. It belongs to the S10E family of ribosomal proteins. It is located in the cytoplasm. |
| UBB | UBB encodes ubiquitin B, a polyubiquitin precursor. Ubiquitin is a highly conserved protein involved in targeting cellular proteins for degradation by the 26S proteosome. |
| RPL17-C18orf32 | RPL17-C18orf32 is a fusion gene between RPL17 and C18orf32. RPL17 encodes a ribosomal protein of the 60S subunit. C18orf32 is a protein coding gene of unknown function. |
| SRP14 | SRP14 encodes a component of the signal recognition particle, which targets specific proteins to the endoplasmic reticulum and is involved in the translocation of nascent proteins. It binds the 7S RNA component of the SRP. |
| RPS10-NUDT3 | RPS10-NUDT3 is a fusion gene between RPS10 and NUDT3. RPS10 encodes a ribosomal protein of the 40S subunit. NUDT3 encodes a protein that hydrolyzes 8-oxo-dGTP to prevent DNA mutations. |
| GAPDH | GAPDH encodes glyceraldehyde-3-phosphate dehydrogenase, an enzyme involved in glycolysis and nuclear functions including transcription, RNA transport, DNA replication and apoptosis. |
| NME2 | NME2 encodes a nucleoside diphosphate kinase involved in the synthesis of nucleoside triphosphates other than ATP. It may act as a transcriptional activator. |
| RPL41 | RPL41 encodes a ribosomal protein that is a component of the 60S subunit. It belongs to the L41E family of ribosomal proteins. |
| HSP90AB1 | HSP90AB1 encodes a heat shock protein that is involved in the proper folding of proteins and degradation of misfolded proteins. It is an ATPase and molecular chaperone. |
| ATP5F1B | ATP5F1B encodes a subunit of mitochondrial ATP synthase, which produces ATP from ADP in the presence of a proton gradient across the membrane. |
| COX7C | COX7C encodes a component of cytochrome c oxidase, the terminal oxidase of the mitochondrial respiratory chain. It transfers electrons from cytochrome c to oxygen. |
| OST4 | OST4 encodes an enzyme that catalyzes the transfer of N-acetylglucosamine to serine residues on specific proteins, as part of glycosylphosphatidylinositol (GPI)-anchor biosynthesis. |
| TPI1 | TPI1 encodes an enzyme that interconverts dihydroxyacetone phosphate and D-glyceraldehyde-3-phosphate in glycolysis and gluconeogenesis. |
| PSMA6 | Diseases associated with PSMA6 include Myocardial Infarction and Combined Oxidative Phosphorylation Deficiency 8. Among its related pathways are Regulation of activated PAK-2p34 by proteasome mediated degradation and Assembly of the pre-replicative complex. Gene Ontology (GO) annotations related to this gene include RNA binding and NF-kappaB binding. An important paralog of this gene is PSMA7 |
| NME1-NME2 | NME1-NME2 is a Protein Coding gene. Diseases associated with NME1-NME2 include Endometrium Carcinoma In Situ and Nemaline Myopathy 2. Among its related pathways are Pyrimidine metabolism and Metabolism of nucleotides. Gene Ontology (GO) annotations related to this gene include DNA-binding transcription factor activity and protein histidine kinase activity |

**Supplementary Table 3: Functional annotations of 10 dark nodes in the first group PPI networks(GeneCards)**

| **Gene** | **Annotations** |
| --- | --- |
| PRDX6 | Thiol-specific peroxidase that catalyzes the reduction of hydrogen peroxide and organic hydroperoxides to water and alcohols, respectively (PubMed:9497358, PubMed:10893423).  Can reduce H2O2 and short chain organic, fatty acid, and phospholipid hydroperoxides (PubMed:10893423).  Also has phospholipase activity, can therefore either reduce the oxidized sn-2 fatty acyl group of phospholipids (peroxidase activity) or hydrolyze the sn-2 ester bond of phospholipids (phospholipase activity) (PubMed:10893423, PubMed:26830860).  These activities are dependent on binding to phospholipids at acidic pH and to oxidized phospholipds at cytosolic pH (PubMed:10893423).  Plays a role in cell protection against oxidative stress by detoxifying peroxides and in phospholipid homeostasis (PubMed:10893423).  Exhibits acyl-CoA-dependent lysophospholipid acyltransferase which mediates the conversion of lysophosphatidylcholine (1-acyl-sn-glycero-3-phosphocholine or LPC) into phosphatidylcholine (1,2-diacyl-sn-glycero-3-phosphocholine or PC) (PubMed:26830860).  Shows a clear preference for LPC as the lysophospholipid and for palmitoyl CoA as the fatty acyl substrate (PubMed:26830860) (224 aa) |
| ETS2 | Protein C-ets-2; Transcription factor activating transcription. Binds specifically the DNA GGAA/T core motif (Ets-binding site or EBS) in gene promoters and stimulates transcription. (469 aa) |
| IFIT3 | Interferon-induced protein with tetratricopeptide repeats 3; IFN-induced antiviral protein which acts as an inhibitor of cellular as well as viral processes, cell migration, proliferation, signaling, and viral replication. Enhances MAVS-mediated host antiviral responses by serving as an adapter bridging TBK1 to MAVS which leads to the activation of TBK1 and phosphorylation of IRF3 and phosphorylated IRF3 translocates into nucleus to promote antiviral transcription. Exihibits an antiproliferative activity via the up regulation of cell cycle negative regulators CDKN1A/p21 and CDKN1B [...] (490 aa) |
| CYB5A | Cytochrome b5; Cytochrome b5 is a membrane-bound hemoprotein functioning as an electron carrier for several membrane-bound oxygenases. (134 aa) |
| GAMT | Guanidinoacetate N-methyltransferase; Converts guanidinoacetate to creatine, using S- adenosylmethionine as the methyl donor. Important in nervous system development. (269 aa) |
| FBXO21 | F-box only protein 21; Substrate-recognition component of the SCF (SKP1-CUL1-F-box protein)-type E3 ubiquitin ligase complex. (628 aa) |
| ACSM3 | Acyl-coenzyme A synthetase ACSM3, mitochondrial; Catalyzes the activation of fatty acids by CoA to produce an acyl-CoA, the first step in fatty acid metabolism (By similarity). Capable of activating medium-chain fatty acids with a preference for isobutyrate among fatty acids with 2-6 carbon atoms (By similarity). (586 aa) |
| SIK1 | Serine/threonine-protein kinase SIK1; Serine/threonine-protein kinase involved in various processes such as cell cycle regulation, gluconeogenesis and lipogenesis regulation, muscle growth and differentiation and tumor suppression. Phosphorylates HDAC4, HDAC5, PPME1, SREBF1, CRTC1/TORC1. Inhibits CREB activity by phosphorylating and inhibiting activity of TORCs, the CREB- specific coactivators, like CRTC2/TORC2 and CRTC3/TORC3 in response to cAMP signaling. Acts as a tumor suppressor and plays a key role in p53/TP53-dependent anoikis, a type of apoptosis triggered by cell detachment: r [...] (783 aa) |
| SPAG4 | Sperm-associated antigen 4 protein; Involved in spermatogenesis. Required for sperm head formation but not required to establish and maintain general polarity of the sperm head. Required for anchoring and organization of the manchette. Required for targeting of SUN3 and probably SYNE1 through a probable SUN1:SYNE3 LINC complex to the nuclear envelope and involved in accurate posterior sperm head localization of the complex. May anchor SUN3 the nuclear envelope. Involved in maintenance of the nuclear envelope integrity. May assist the organization and assembly of outer dense fibers (ODF [...] (437 aa) |
| JUNB | Transcription factor jun-B; Transcription factor involved in regulating gene activity following the primary growth factor response. Binds to the DNA sequence 5'-TGA[CG]TCA-3'; Belongs to the bZIP family. Jun subfamily. (347 aa) |

**Supplementary Table 4: Functional annotations of 16 dark nodes in the second group PPI networks(GeneCards)**

| **Gene** | **Annotations** |
| --- | --- |
| RPS27 | 40S ribosomal protein S27; Component of the small ribosomal subunit. Required for proper rRNA processing and maturation of 18S rRNAs; Belongs to the eukaryotic ribosomal protein eS27 family. (84 aa) |
| RPL36 | 60S ribosomal protein L36; Component of the large ribosomal subunit. (105 aa) |
| SF3B5 | Splicing factor 3B subunit 5; Involved in pre-mRNA splicing as a component of the splicing factor SF3B complex, a constituent of the spliceosome. SF3B complex is required for 'A' complex assembly formed by the stable binding of U2 snRNP to the branchpoint sequence (BPS) in pre-mRNA. Sequence independent binding of SF3A/SF3B complex upstream of the branch site is essential, it may anchor U2 snRNP to the pre-mRNA. (86 aa) |
| NME2 | Nucleoside diphosphate kinase B; Major role in the synthesis of nucleoside triphosphates other than ATP. The ATP gamma phosphate is transferred to the NDP beta phosphate via a ping-pong mechanism, using a phosphorylated active-site intermediate (By similarity). Negatively regulates Rho activity by interacting with AKAP13/LBC. Acts as a transcriptional activator of the MYC gene; binds DNA non-specifically. Binds to both single-stranded guanine- and cytosine-rich strands within the nuclease hypersensitive element (NHE) III(1) region of the MYC gene promoter. Does not bind to duplex NHE I [...] (152 aa) |
| RPS14 | Ribosomal protein S14. (151 aa) |
| RPL30 | Ribosomal protein L30. (115 aa) |
| TFPI | Tissue factor pathway inhibitor: Inhibits factor X (X(a)) directly and, in a Xa-dependent way, inhibits VIIa/tissue factor activity, presumably by forming a quaternary Xa/LACI/VIIa/TF complex. It possesses an antithrombotic action and also the ability to associate with lipoproteins in plasma. (304 aa) |
| RPL17 | 60S ribosomal protein L17; Component of the large ribosomal subunit. Belongs to the universal ribosomal protein uL22 family. (184 aa) |
| RPS11 | Ribosomal protein S11; Belongs to the universal ribosomal protein uS17 family. (158 aa) |
| RPL24 | Ribosomal protein L24; Belongs to the eukaryotic ribosomal protein eL24 family. (157 aa) |
| RPL38 | Ribosomal protein L38; Belongs to the eukaryotic ribosomal protein eL38 family. (70 aa) |
| TMSB10 | Thymosin beta-10; Plays an important role in the organization of the cytoskeleton. Binds to and sequesters actin monomers (G actin) and therefore inhibits actin polymerization (By similarity); Belongs to the thymosin beta family. (44 aa) |
| RPS5 | 40S ribosomal protein S5, N-terminally processed; Ribosomal protein S5; Belongs to the universal ribosomal protein uS7 family. (204 aa) |
| ZYX | Zyxin; Adhesion plaque protein. Binds alpha-actinin and the CRP protein. Important for targeting TES and ENA/VASP family members to focal adhesions and for the formation of action-rich structures. May be a component of a signal transduction pathway that mediates adhesion- stimulated changes in gene expression (By similarity); Belongs to the zyxin/ajuba family. (572 aa) |
| TUBA1C | Detyrosinated tubulin alpha-1C chain; Tubulin is the major constituent of microtubules. It binds two moles of GTP, one at an exchangeable site on the beta chain and one at a non-exchangeable site on the alpha chain; Belongs to the tubulin family. (519 aa) |
| CETN2 | Centrin-2; Plays a fundamental role in microtubule organizing center structure and function. Required for centriole duplication and correct spindle formation. Has a role in regulating cytokinesis and genome stability via cooperation with CALM1 and CCP110. The XPC complex is proposed to represent the first factor bound at the sites of DNA damage and together with other core recognition factors, XPA, RPA and the TFIIH complex, is part of the pre-incision (or initial recognition) complex. The XPC complex recognizes a wide spectrum of damaged DNA characterized by distortions of the DNA hel [...] (172 aa) |

**Supplementary Table 5: Functional and pathway enrichment of co-DEGs in the first group (STRING)**

| **GO-term** | **Description** | **Count in Network** | **Strength** | **FDR** |
| --- | --- | --- | --- | --- |
| BP-GO:0048545 | Response to steroid hormone | 10 of 285 | 0.89 | 0.0131 |
| CC-GO:0031012 | Extracellular matrix | 17 of 552 | 0.83 | 1.13e-06 |
| CC-GO:0062023 | Collagen-containing extracellular matrix | 15 of 407 | 0.91 | 1.13e-06 |
| KEGG-hsa04668 | TNF signaling pathway | 7 of 111 | 1.14 | 0.00035 |

**Supplementary Table 6: Functional enrichment of co-DEGs in the second group (STRING)**

| **GO Term** | **Category** | **Description** | **Count in network** | **Strength** | **FDR** |
| --- | --- | --- | --- | --- | --- |
| GO:0002181 | BP | Cytoplasmic translation | 39 of 123 | 1.81 | 3.80 e-53 |
| GO:0000028 | BP | Ribosomal small subunit assembly | 6 of 19 | 1.81 | 9.87 e-07 |
| GO:0006228 | BP | UTP biosynthetic process | 3 of 10 | 1.79 | 0.0098 |
| GO:0006183 | BP | GTP biosynthetic process | 3 of 11 | 1.75 | 0.0119 |
| GO:0006241 | BP | CTP biosynthetic process | 3 of 14 | 1.64 | 0.0208 |
| GO:0000027 | BP | Ribosomal large subunit assembly | 4 of 25 | 1.52 | 0.0039 |
| GO:0042255 | BP | Ribosome assembly | 9 of 60 | 1.49 | 2.83 e-08 |
| GO:0042273 | BP | Ribosomal large subunit biogenesis | 9 of 74 | 1.4 | 1.44 e-07 |
| GO:0006412 | BP | Translation | 43 of 389 | 1.36 | 4.85 e-42 |
| GO:0042274 | BP | Ribosomal small subunit biogenesis | 8 of 78 | 1.32 | 4.48 e-06 |
| GO:0006165 | BP | Nucleoside diphosphate phosphorylation | 6 of 63 | 1.29 | 0.00047 |
| GO:0006414 | BP | Translational elongation | 4 of 47 | 1.24 | 0.0300 |
| GO:0034645 | BP | Cellular macromolecule biosynthetic process | 45 of 778 | 1.07 | 4.09 e-33 |
| GO:0022618 | BP | Ribonucleoprotein complex assembly | 11 of 203 | 1.05 | 3.76 e-06 |
| GO:0042254 | BP | Ribosome biogenesis | 16 of 299 | 1.04 | 1.82 e-09 |
| GO:0009206 | BP | Purine ribonucleoside triphosphate biosynthesis | 5 of 98 | 1.02 | 0.0386 |
| GO:0003735 | MF | Structural constituent of ribosome | 40 of 169 | 1.69 | 8.89e-51 |
| GO:0019843 | MF | rRNA binding | 7 of 67 | 1.33 | 4.92e-05 |
| GO:0005198 | MF | Structural molecule activity | 46 of 776 | 1.09 | 1.66e-34 |
| GO:0097452 | CC | GAIT complex | 2 of 4 | 2.01 | 0.0191 |
| GO:0022625 | CC | Cytosolic large ribosomal subunit | 25 of 56 | 1.96 | 8.09 e-37 |
| GO:0022626 | CC | Cytosolic ribosome | 40 of 101 | 1.91 | 7.61 e-59 |
| GO:0098556 | CC | Cytoplasmic side of rough endoplasmic reticulum | 2 of 5 | 1.91 | 0.0253 |
| GO:0042788 | CC | Polysomal ribosome | 12 of 31 | 1.9 | 2.31 e-16 |
| GO:0022627 | CC | Cytosolic small ribosomal subunit | 16 of 44 | 1.87 | 9.28 e-22 |
| GO:0005844 | CC | Polysome | 15 of 65 | 1.68 | 7.20 e-18 |
| GO:0015934 | CC | Large ribosomal subunit | 26 of 113 | 1.67 | 4.06 e-32 |
| GO:0044391 | CC | Ribosomal subunit | 41 of 187 | 1.65 | 1.06 e-51 |
| GO:1990904 | CC | Ribonucleoprotein complex | 46 of 687 | 1.14 | 1.90 e-37 |
| GO:0005925 | CC | Focal adhesion | 22 of 416 | 1.04 | 2.32 e-14 |

**Supplementary Table 7: KEGG pathway enrichment of co-DEGs in the second group (STRING)**

| **Pathway** | **Description** | **Count in Network** | **Strength** | **False Discovery Rate** |
| --- | --- | --- | --- | --- |
| hsa03010 | Ribosome | 39 of 131 | 1.79 | 6.73e-54 |
| hsa05012 | Parkinson disease | 10 of 236 | 0.94 | 5.44e-05 |
| hsa00190 | Oxidative phosphorylation | 5 of 128 | 0.9 | 0.0228 |
| hsa05020 | Prion disease | 8 of 263 | 0.8 | 0.0058 |
| hsa05016 | Huntington disease | 8 of 295 | 0.75 | 0.0076 |
| hsa05010 | Alzheimer disease | 9 of 354 | 0.72 | 0.0058 |
| hsa05014 | Amyotrophic lateral sclerosis | 8 of 350 | 0.67 | 0.0197 |

**Supplementary Table 8: Full names and aliases of the top 10 Hub genes in the first group (GeneCards)**

| **Genes** | **Full names** | **Aliases** |
| --- | --- | --- |
| ATF3 | activating transcription factor 3 |  |
| COL1A1 | collagen type I alpha 1 chain | CAFYD, EDSARTH1, EDSC, O11, OI2, OI3, O14 |
| CXCR4 | C-X-C motif chemokine receptor 4 | CD184, D2S201E, FB22, HM89, HSY3RR, LAP-3, LAP3, LCR1, LESTR, NPY3R, NPYR, NPYRL, NPYY3R, WHIM, WHIMS, WHIMS1 |
| FOSB | FosB proto-oncogene, AP-1 transcription factor subunit | AP-1, GOS 3, GOSB |
| JUNB | JunB proto-oncogene, AP-1 transcription factor subunit | AP-1 |
| NR4A1 | nuclear receptor subfamily 4 group A member 1 | GFRP1, HMR, N10, NAK-1, NGFIB, NP10, NUR77, TR3 |
| NR4A3 | nuclear receptor subfamily 4 group A member 3 | CHN, CSMF, MINOR, NOR1 |
| PTGS2 | prostaglandin-endoperoxide synthase 2 | COX-2, COX2, GRIPGHS, PGG/HS, PGHS-2, PHS-2, hCox-2 |
| SIK1 | salt inducible kinase 1 | DEE30, MSK, SIK, SIK-1, SIK1B, SNF1LK |
| THBS1 | thrombospondin 1 | THBS, THBS-1, TSP, TSP-1, TSP1 |

**Supplementary Table 9: Full names and aliases of the top 10 Hub genes in the second group (GeneCards)**

| **Symbol** | **Full Name** | **Aliases** |
| --- | --- | --- |
| EEF1B2 | eukaryotic translation elongation factor 1 beta 2 | EEF1B, EEF1B1, EF1B |
| EEF1G | eukaryotic translation elongation factor 1 gamma | EF1G, GIG35 |
| RPL13A | ribosomal protein L13a | L13A, TSTA1, uL13 |
| RPL9 | ribosomal protein L9 | L9, NPC-A-16, uL6 |
| RPLPO | ribosomal protein lateral stalk subunit P0 | L10E, LP0, P0, PRLP0, RPP0, uL10 |
| RPS14 | ribosomal protein S14 | EMTB, S14, uS11 |
| RPS16 | ribosomal protein S16 | S16, uS9 |
| RPS2 | ribosomal protein S2 | LLREP3, S2, uS5 |
| RPSA | ribosomal protein SA | 37LRP, 67LR, ICAS, LAMBR, LAMR1, LBP, LBP/p40, LRP/LR, NEM/1CHD4, SA, lamR, p40, uS2 |
| UBA52 | ubiquitin A-52 residue ribosomal protein fusion product 1 | CEP52, HUBCEP52, L40, RPL40 |

**Supplementary Table 10: Top 17 high-scored pathways of the first group's top 10 Hub genes (GeneCards)**

| **Scores** | **SuperPath name** |
| --- | --- |
| 24.03 | Toll Comparative Pathway |
| 21.46 | IL-1 Family Signaling Pathways |
| 20.49 | Tacrolimus/Cyclosporine Pathway, Pharmacodynamics |
| 19.12 | TCR Signaling (Qiagen) |
| 18.79 | ERK Signaling |
| 18.55 | AP-1 Transcription Factor Network |
| 17.36 | Corticotropin-releasing Hormone Signaling Pathway |
| 17.31 | NF-kappaB Signaling |
| 17.27 | Ceramide Pathway |
| 16.62 | IL-17 Family Signaling Pathways |
| 16.13 | SMAD Signaling Network |
| 15.30 | Development VEGF Signaling Via VEGFR2 - Generic Cascades |
| 15.24 | CNTF Signaling |
| 15.04 | Hypertrophy Model |
| 14.65 | Signaling By Receptor Tyrosine Kinases |
| 13.78 | Syndecan-4-mediated Signaling Events |
| 13.60 | Inflammatory Response Pathway |

**Supplementary Table 11: Top 14 high-scored pathways of the second group's top 10 Hub genes (GeneCards)**

| **Score** | **Super-Path Name** |
| --- | --- |
| 58.83 | Viral MRNA Translation |
| 53.00 | Influenza Infection |
| 49.83 | RRNA Processing in The Nucleus and Cytosol |
| 40.44 | Regulation of Expression of SLITs and ROBOs |
| 36.43 | Metabolism of Proteins |
| 34.95 | Processing of Capped Intron-Containing Pre-mRNA |
| 34.11 | Cellular Responses to Stimuli |
| 31.12 | Infectious Disease |
| 29.93 | Nervous System Development |
| 29.24 | SARS-CoV-1-host Interactions |
| 27.74 | SARS-CoV-2 Modulates Host Translation Machinery |
| 26.93 | Activation of The MRNA Upon Binding of The Cap-binding Complex and EIFs, and Subsequent Binding to 43 S |
| 23.03 | Metabolism |
| 21.59 | SARS-CoV-2 Infection |

**Supplementary Table 12: Top 21 high-scored GO annotations for the first group's top 10 Hub genes (GeneCards)**

| **Score** | **Name** | **Ontology** |
| --- | --- | --- |
| 20.54 | DNA-binding Transcription Activator Activity, RNA Polymerase II-specific | MF |
| 19.45 | Positive Regulation of Smooth Muscle Cell Proliferation | BP |
| 19.04 | Cellular Response to Corticotropin-releasing Hormone Stimulus | BP |
| 19.01 | Response to Mechanical Stimulus | BP |
| 18.68 | Negative Regulation of Cysteine-type Endopeptidase Activity Involved in Apoptotic Process | BP |
| 18.47 | Response to Xenobiotic Stimulus | BP |
| 17.98 | DNA-binding Transcription Factor Activity | MF |
| 17.35 | Positive Regulation of Transforming Growth Factor Beta Production | BP |
| 17.35 | Regulation of Type B Pancreatic Cell Proliferation | BP |
| 16.29 | Nuclear Glucocorticoid Receptor Binding | MF |
| 15.54 | Response to Organic Cyclic Compound | BP |
| 15.19 | Positive Regulation of Chemotaxis | BP |
| 14.78 | Chromatin | CC |
| 14.40 | Response to Morphine | BP |
| 14.29 | Decidualization | BP |
| 14.23 | Sequence-specific Double-stranded DNA Binding | MF |
| 13.99 | RNA Polymerase II Cis-regulatory Region Sequence-specific DNA Binding | MF |
| 13.96 | Positive Regulation of Transcription By RNA Polymerase II | BP |
| 13.75 | DNA-binding Transcription Factor Activity, RNA Polymerase II-specific | MF |
| 13.69 | Cellular Response to Fibroblast Growth Factor Stimulus | BP |
| 13.60 | Response to Progesterone | BP |

**Supplementary Table 13: Top 18 high-scored GO annotations for the second group's top 10 Hub genes (GeneCards)**

| **Score** | **Name** | **Ontology** |
| --- | --- | --- |
| 60.95 | Translation | BP |
| 59.54 | Cytosolic Ribosome | CC |
| 59.41 | Cytoplasmic Translation | BP |
| 41.81 | Ribonucleoprotein Complex | CC |
| 37.04 | Cytosolic Small Ribosomal Subunit | CC |
| 36.00 | Structural Constituent of Ribosome | MF |
| 35.54 | Ribosome | CC |
| 27.39 | Focal Adhesion | CC |
| 27.12 | Cytosolic Large Ribosomal Subunit | CC |
| 22.14 | Small Ribosomal Subunit | CC |
| 21.53 | Cytosol | CC |
| 21.08 | RNA Binding | MF |
| 17.76 | Extracellular Exosome | CC |
| 17.28 | Cytoplasm | CC |
| 15.69 | Ribosomal Small Subunit Assembly | BP |
| 14.18 | Translation Elongation Factor Activity | MF |
| 14.08 | Maturation of SSU-rRNA From Tricistronic RRNA Transcript (SSU- rRNA, 5.8S RRNA, LSU-rRNA) | BP |
| 13.88 | Translational Elongation | BP |
